# Supplementary material for: Immunological and clinicopathological features predict HER2-positive breast cancer prognosis in the neoadjuvant NeoALTTO and CALGB 40601 randomized trials
Source: Nat Commun. 2023 Nov 3;14:7053. doi: 10.1038/s41467-023-42635-2 (PMC10624889; doi:10.1038/s41467-023-42635-2)
Supplement: Supplementary file 1 — Supplementary Information [file 41467_2023_42635_MOESM1_ESM.pdf]

Immunological and clinicopathological features predict HER2-positive breast cancer prognosis in the neoadjuvant NeoALTTO and CALGB 40601 randomized trials

Authors:

Mattia Rediti[1], Aranzazu Fernandez-Martinez[2], David Venet[1], Françoise Rothé[1], Katherine A Hoadley[2], Joel S Parker[2], Baljit Singh[3], Jordan D Campbell[4], Karla V Ballman[5], David W Hillman[4], Eric P Winer[6], Sarra El-Abed[7], Martine Piccart[8], Serena Di Cosimo[9], William Fraser Symmans[10], Ian E Krop[6], Roberto Salgado[11,12], Sherene Loi[12], Lajos Pusztai[13], Charles M Perou[2], Lisa A Carey[14], Christos Sotiriou[1].

Affiliations:

[1] Breast Cancer Translational Research Laboratory, Institut Jules Bordet, Hôpital Universitaire de Bruxelles (H.U.B), Université Libre de Bruxelles (ULB), Brussels, Belgium
[2] Lineberger Comprehensive Cancer Center, University of North Carolina, Chapel Hill, NC, USA
[3] White Plains Hospital, White Plains, NY, USA
[4] Alliance Statistics and Data Management Center, Mayo Clinic, Rochester, MN, USA
[5] Alliance Statistics and Data Management Center, Weill Cornell Medicine, New York, NY, USA
[6] Yale Cancer Center, Yale School of Medicine, New Haven, CT, USA
[7] Breast International Group, Brussels, Belgium
[8] Medical Oncology Department, Institut Jules Bordet and l'Université Libre de Bruxelles (U.L.B.), Brussels, Belgium.
[9] Integrated biology platform unit, Department of Applied Research and Technological Development, Fondazione IRCCS Istituto Nazionale dei Tumori, Milano
[10] Department of Pathology, University of Texas, MD Anderson Cancer Center, Houston, TX 77030, USA
[11] Department of Pathology, GZA-ZNA Ziekenhuizen, Antwerp, Belgium
[12] Division of Research, Peter MacCallum Cancer Centre, Melbourne, Australia
[13] Breast Medical Oncology, Yale Cancer Center, Yale School of Medicine, New Haven, CT, USA
[14] Division of Hematology-Oncology, Department of Medicine, School of Medicine, University of North Carolina at Chapel Hill, Chapel Hill, NC, USA

This file contains:

- All supplementary Figures (1 to 25)

- Supplementary Tables 1 and 2

SUPPLEMENTARY FIGURES

Supplementary Figure 1

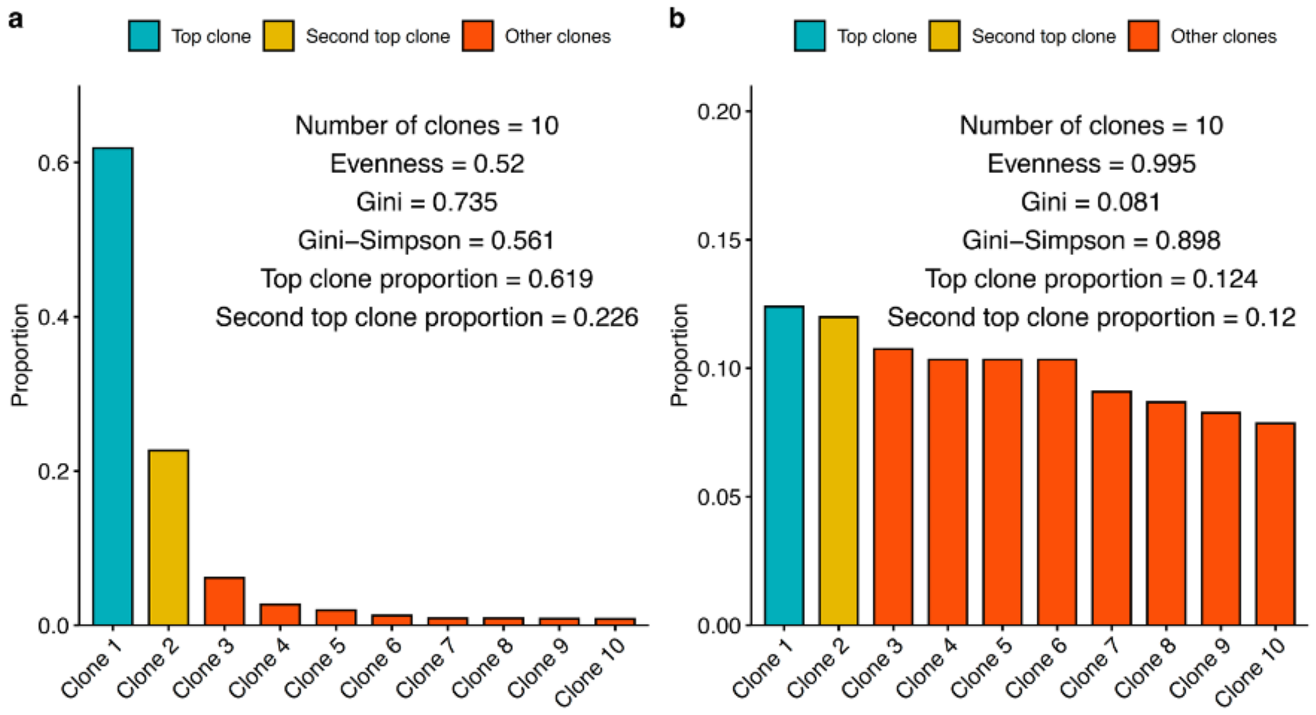

Illustrative examples of hypothetical BCR/TCR repertoires characterized by populations presenting different degrees of clonal expansion.

In these examples, two samples presenting 10 different clones are shown; top and second top clone proportions measure the proportion of reads mapping to the first and second most expressed clone for that sample; Gini index describes the degree of inequality among the population; Gini-Simpson index describes the probability that the two entities represent different types; evenness measures distribution equality.

a BCR/TCR repertoire characterized by clonal expansion and a more antigen-specific immune response. Here, one clone (top clone) is predominant, while other clones are present at lower frequencies. Evenness and Gini index present low and high values, respectively.

b BCR/TCR repertoire characterized by lack of clonal expansion and a more heterogeneous immune response. Here, no clone is predominant, and frequencies are similar across the clonal populations, as also described by higher evenness and lower Gini index.

Data to recreate the two illustrative examples are available at https://github.com/BCTL-Bordet/BCR_TCR_analyses.

BCR: B cell receptor; TCR: T cell receptor.

Supplementary Figure 2

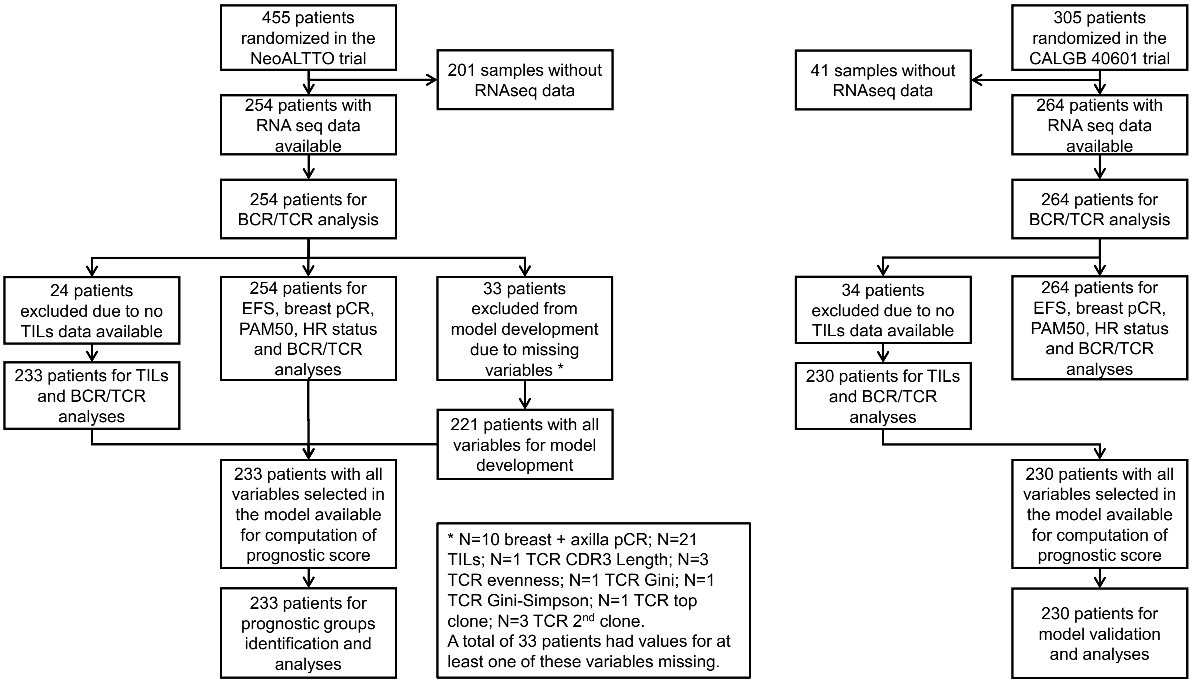

Consort diagrams showing patients selected from the NeoALTTO and CALGB 40601 RNA sequencing cohorts for downstream analyses.

In NeoALTTO, 254 patients had RNA sequencing data available. Among those, 233 had TILs data as well. The prognostic model was developed on a cohort of 221 patients with all variables tested available. A cohort of 233 patients, with all variables selected from the model, was used to calculate the prognostic score derived from the model, including cut-off identification for the prognostic groups and downstream analyses. In CALGB 40601, 264 patients had RNA sequencing data available, among whom 230 also had TILs data. This cohort was used to validate the prognostic model and to perform downstream analyses.

BCR: B cell receptor; CDR3: complementarity-determining region 3; HR: hormone receptor; pCR: pathological complete response; TCR: T cell receptor; TILs: tumor-infiltrating lymphocytes.

Supplementary Figure 3

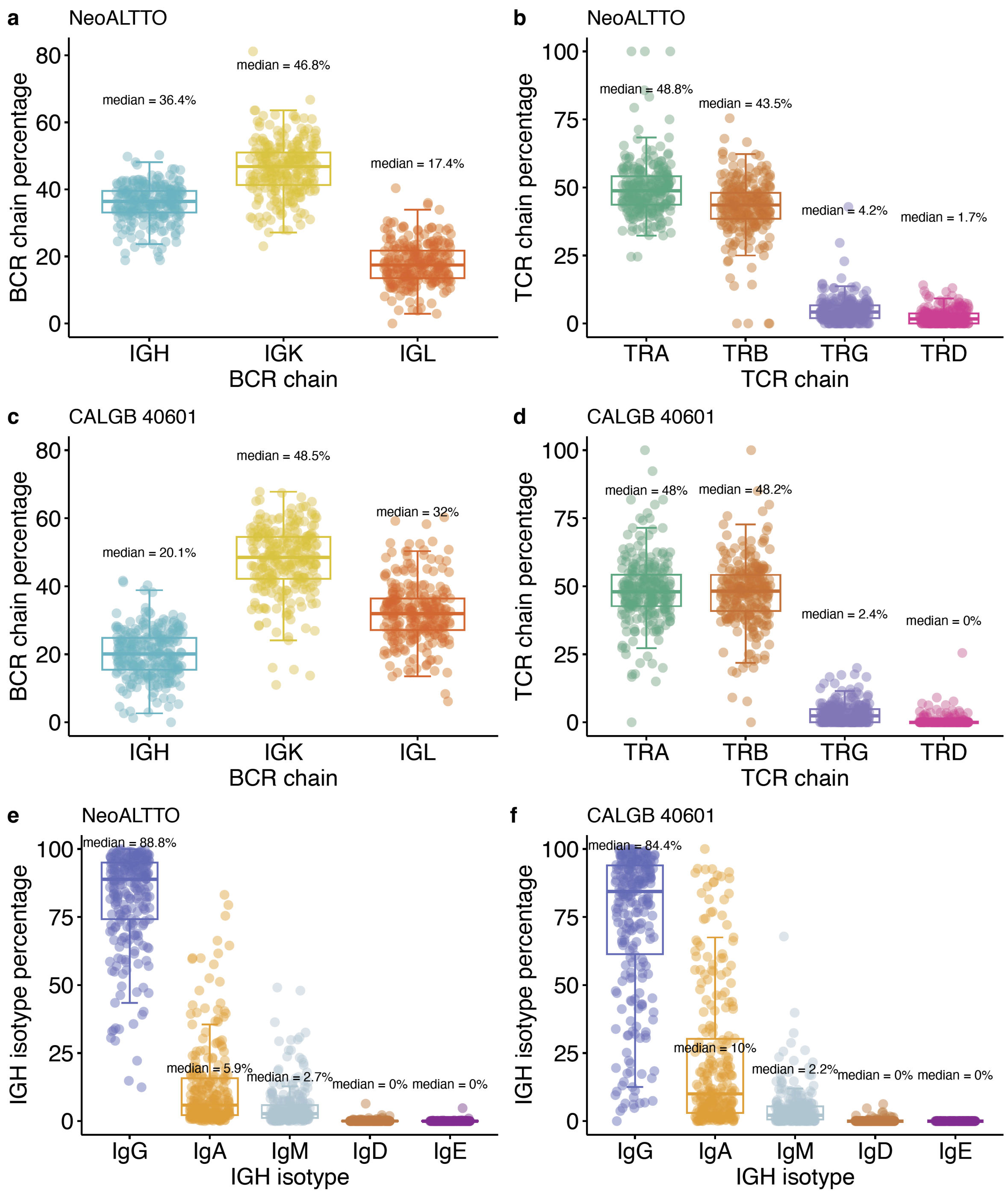

Proportions of reads mapping to immunoglobulin chains/isotypes, and TCR chains in NeoALTTO and CALGB 40601.

a Proportions of IGH, IGK and IGL in NeoALTTO (N = 254 with at least 1 BCR read). **b** Proportions of TRA, TRB, TRG and TRD in NeoALTTO (N = 253 with at least 1 TCR read). **c** Proportions of IGH, IGK and IGL in CALGB 40601 (N = 264 with at least 1 BCR read). **d** Proportions of TRA, TRB, TRG and TRD in CALGB 40601 (N = 264 with at least 1 TCR read). **e** Proportions of IGH isotypes in NeoALTTO (N = 254). **f** Proportions of IGH isotypes in CALGB 40601 (N = 262 with at least 1 IGH read and considering IGH clones for which the constant region information was available; in 1 sample there were more than 1 IGH read, but the information about the constant region was not available, while in another one there were no IGH reads).

Proportions are calculated after excluding BCR/TCR clones for which chain and/or isotype information could not be computed (details in **METHODS**). In boxplots, the boxes are defined by

the upper and lower quartile; the median is shown as a bold colored horizontal line; whiskers extend to the most extreme data point which is no more than 1.5 times the interquartile range from the box. Median values are reported. Source data are available.

BCR: B cell receptor; Ig: immunoglobulin; IGH: immunoglobulin heavy chain; IGK: immunoglobulin light chain K; IGL: immunoglobulin light chain L; TCR: T cell receptor; TRA: T cell receptor alpha chain; TRB: T cell receptor beta chain; TRD: T cell receptor delta chain; TRG: T cell receptor gamma chain.

Supplementary Figure 4

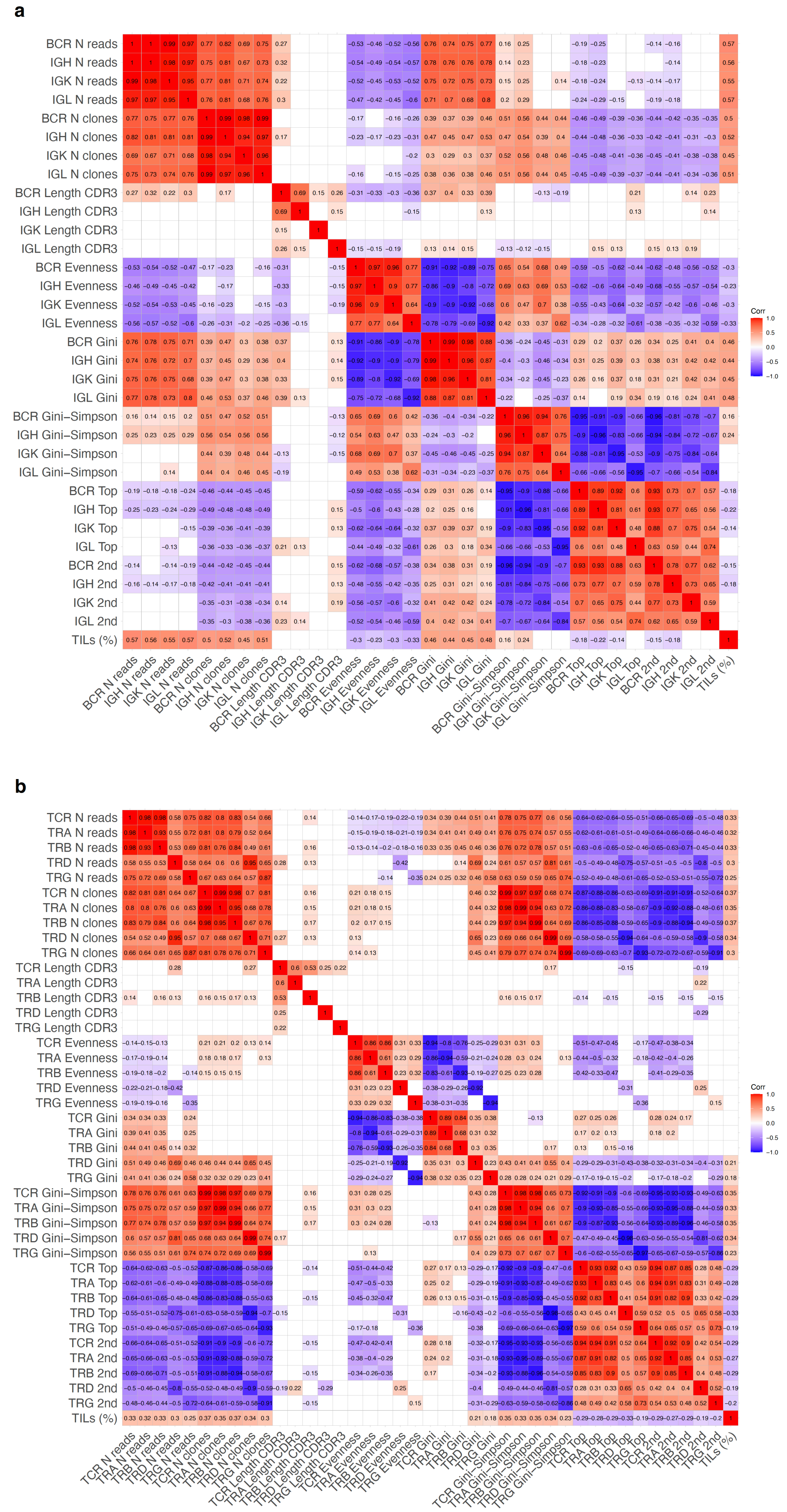

Correlations between single BCR/TCR chains and total BCR/TCR measures derived from all reads mapping to the chains, as well as between different BCR/TCR repertoire measures and TIL levels in NeoALTTO.

a Correlations between BCR chains (IGH, IGK, IGL) and total BCR measures.

b Correlations between TCR chains (TRA, TRB, TRD, TRG) and total TCR measures.

Correlations are Spearman (pairwise complete observations). Only correlations with $P < 0.05$ are shown. Correlations and P values are available in Supplementary data 4. The number of reads (N reads) is normalized by the total number of reads mapping to the transcriptome in each sample, and multiplied by 1000.

BCR: B cell receptor; CDR3: complementarity-determining region 3; IGH: immunoglobulin heavy chain; IGK: immunoglobulin light chain kappa; IGL: immunoglobulin light chain lambda; TCR: T cell receptor; TILs: tumor-infiltrating lymphocytes; TRA: T cell receptor alpha chain; TRB: T cell receptor beta chain; TRD: T cell receptor delta chain; TRG: T cell receptor gamma chain.

Supplementary Figure 5

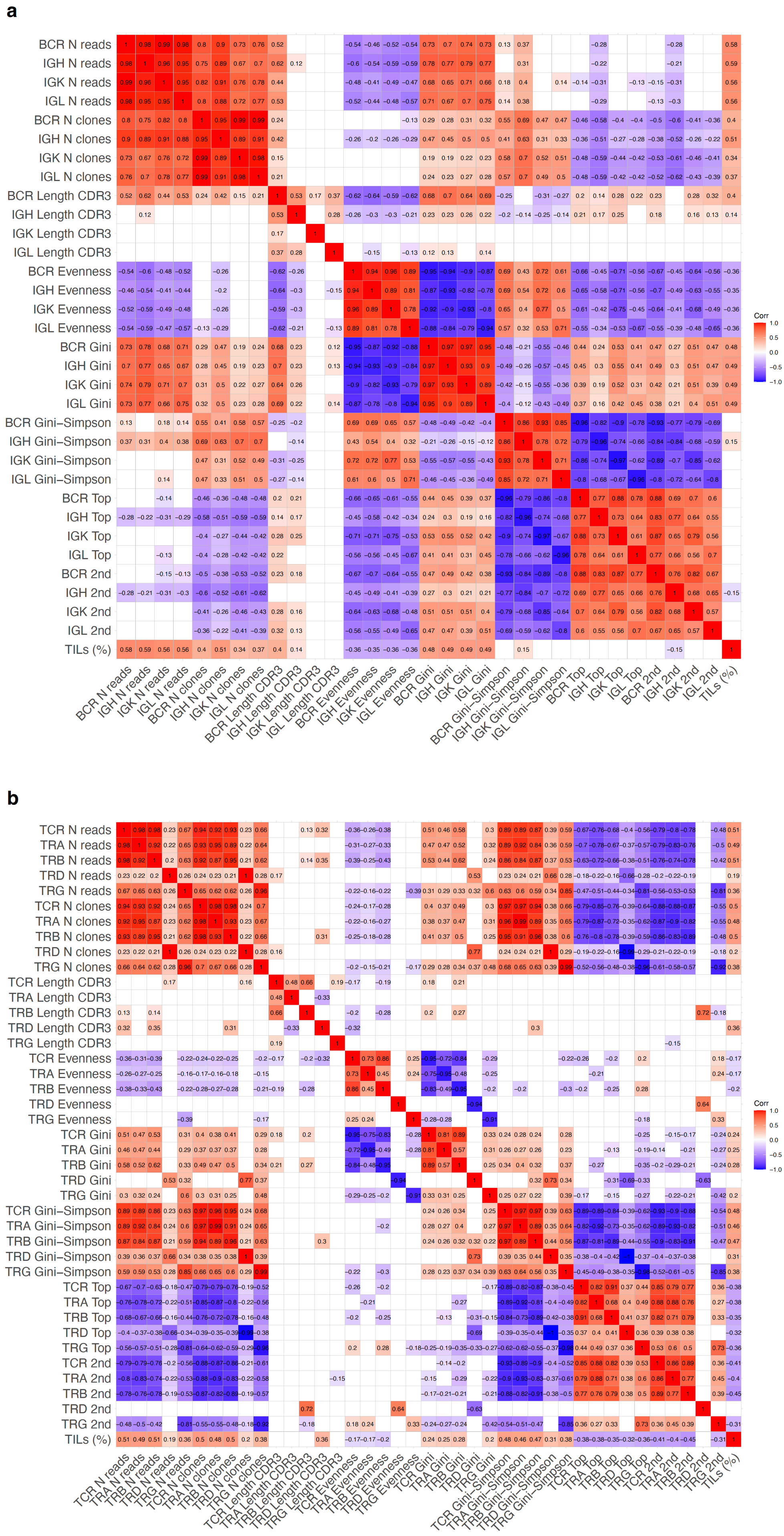

Correlations between single BCR/TCR chains and total BCR/TCR measures derived from all reads mapping to the chains, as well as between different BCR/TCR repertoire measures and TIL levels in CALGB 40601.

a Correlations between BCR chains (IGH, IGK, IGL) and total BCR measures.

b Correlations between TCR chains (TRA, TRB, TRD, TRG) and total TCR measures.

Correlations are Spearman (pairwise complete observations). Only correlations with P < 0.05 are shown. Correlations and P values are available in Supplementary data 5. The number of reads (N reads) is normalized by the total number of reads mapping to the transcriptome in each sample, and multiplied by 1000.

BCR: B cell receptor; CDR3: complementarity-determining region 3; IGH: immunoglobulin heavy chain; IGK: immunoglobulin light chain kappa; IGL: immunoglobulin light chain lambda; TCR: T cell receptor; TILs: tumor-infiltrating lymphocytes; TRA: T cell receptor alpha chain; TRB: T cell receptor beta chain; TRD: T cell receptor delta chain; TRG: T cell receptor gamma chain.

Supplementary Figure 6

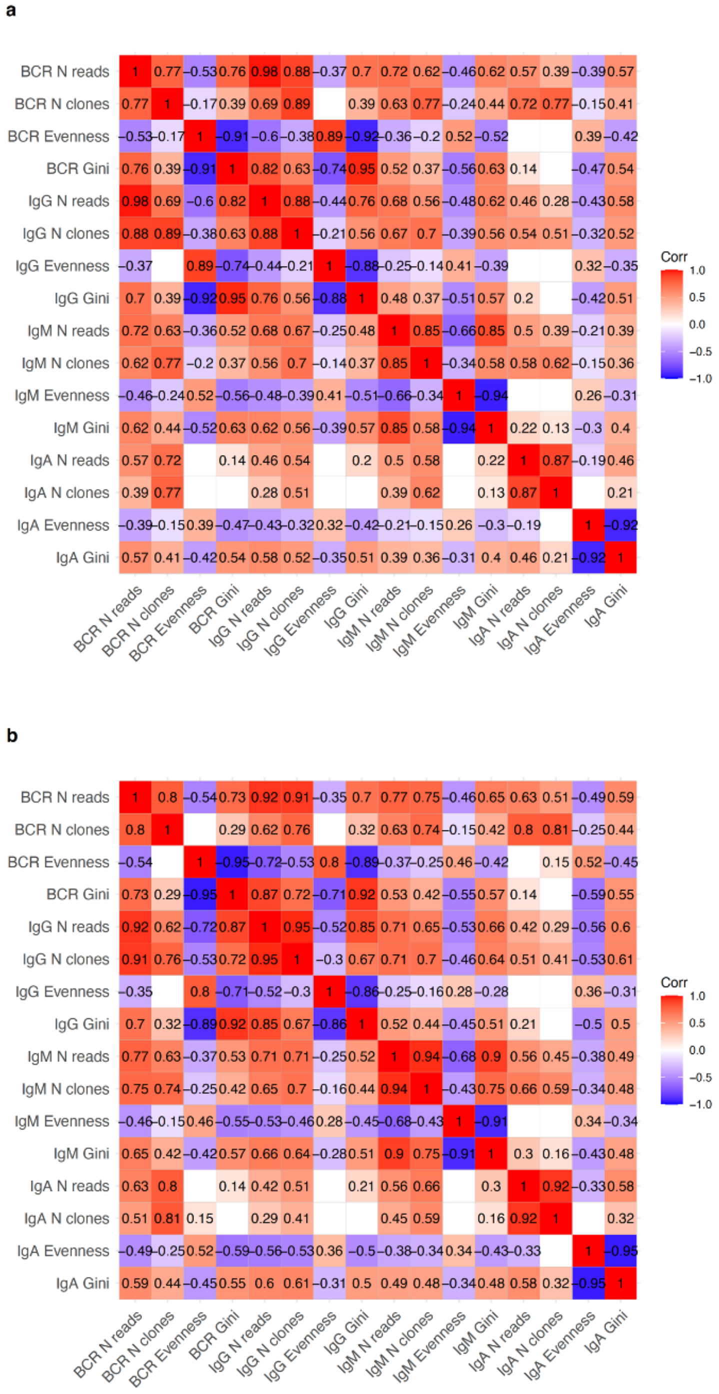

Correlations between selected global BCR, IgG, IgM, and IgA measures in NeoALTTO and CALGB 40601.

a Correlations between total BCR measures and IgG, IgM and IgA isotype measures in NeoALTTO.

b Correlations between total BCR measures and IgG, IgM and IgA isotype measures in CALGB 40601.

Correlations are Spearman (pairwise complete observations). Only correlations with $P < 0.05$ are shown. Correlations and P values are available in Supplementary data 6 (a) and 7 (b). The number of reads (N reads) is normalized by the total number of reads mapping to the transcriptome in each sample, and multiplied by 1000.

BCR: B cell receptor; Ig: immunoglobulin.

Supplementary Figure 7

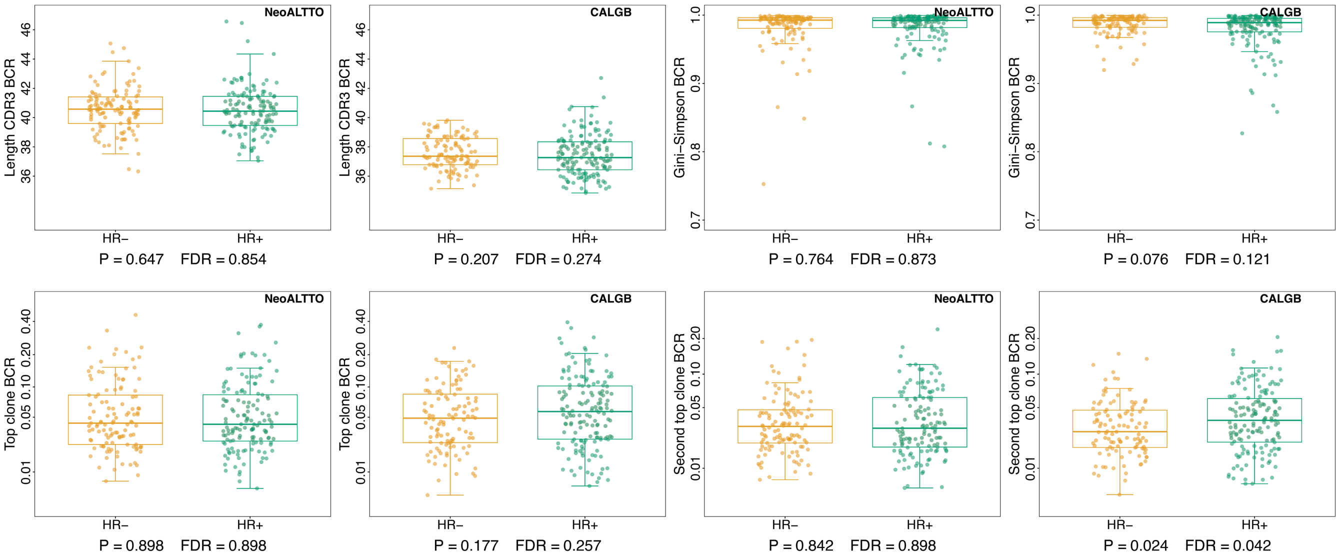

Additional BCR measures according to hormone receptor status.

Comparisons of BCR length of CDR3 (“Length CDR3”), Gini-Simpson, top clone proportion (represented on a log scale) and second top clone proportion (represented on a log scale) in HR- and HR+ HER2-positive breast cancer in NeoALTTO (N = 254) and CALGB 40601 (N = 264). Statistical differences were assessed using Wilcoxon rank sum test (P values at the bottom of the panels), and FDRs obtained adjusting P values using Benjamini & Hochberg method (applied on the comparisons performed for all BCR measures in each study, separately). See also Figure 1. P values and FDRs are available in Supplementary data 8.

In boxplots, the boxes are defined by the upper and lower quartile; the median is shown as a bold colored horizontal line; whiskers extend to the most extreme data point which is no more than 1.5 times the interquartile range from the box.

BCR: B cell receptor; CDR3: complementarity-determining region 3; FDR: false discovery rate; HR: hormone receptor.

Supplementary Figure 8

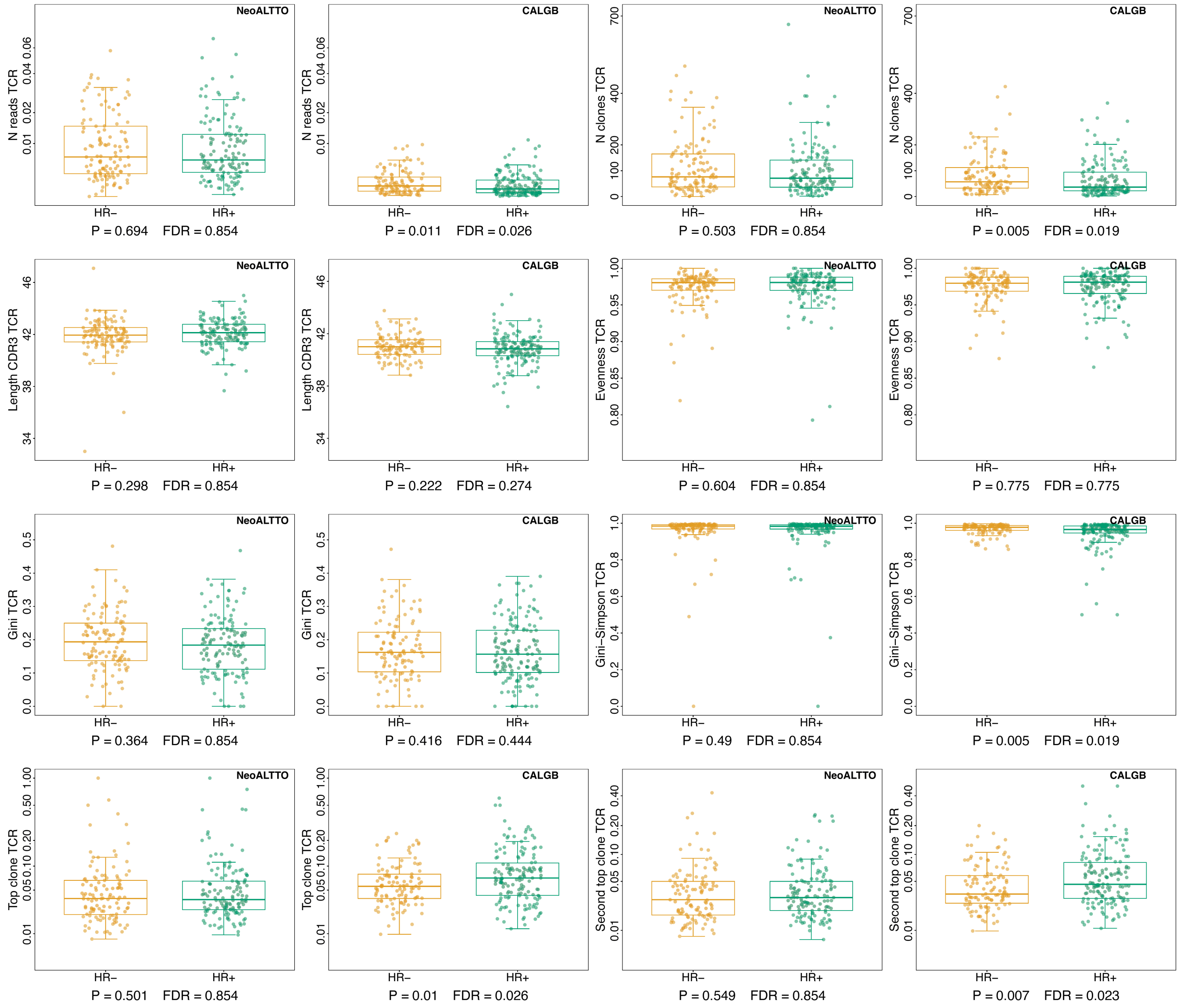

TCR measures according to hormone receptor status.

Comparisons of TCR normalized number of reads ("N reads"; represented on a log scale), number of clones ("N clones"), length of CDR3 ("Length CDR3 T"), evenness, Gini index, Gini-Simpson index, top clone proportion (represented on a log scale) and second top clone proportion (represented on a log scale) in HR- and HR+ HER2-positive breast cancer in NeoALTTO (N = 254, among which 1 had 0 reads mapping to TCR) and CALGB 40601 (N = 264). In NeoALTTO, evenness and second top clone proportion were not calculated in N = 3 (<2 TCR clones present), while length of CDR3, Gini, Gini-Simpson and top clone proportion were not calculated in N = 1 (no TCR clones present). Statistical differences were assessed using Wilcoxon rank sum test (P values at the bottom of the panels), and FDRs obtained adjusting P values using Benjamini & Hochberg method (applied on the comparisons performed for all TCR measures in each study, separately). P values and FDRs are available in Supplementary data 8. P values are two-sided. The number of reads is normalized by the total number of reads mapping to the transcriptome in each sample, and multiplied by 1000.

In boxplots, the boxes are defined by the upper and lower quartile; the median is shown as a bold colored horizontal line; whiskers extend to the most extreme data point which is no more than 1.5 times the interquartile range from the box.

CDR3: complementarity-determining region 3; FDR: false discovery rate; HR: hormone receptor; TCR: T cell receptor.

Supplementary Figure 9

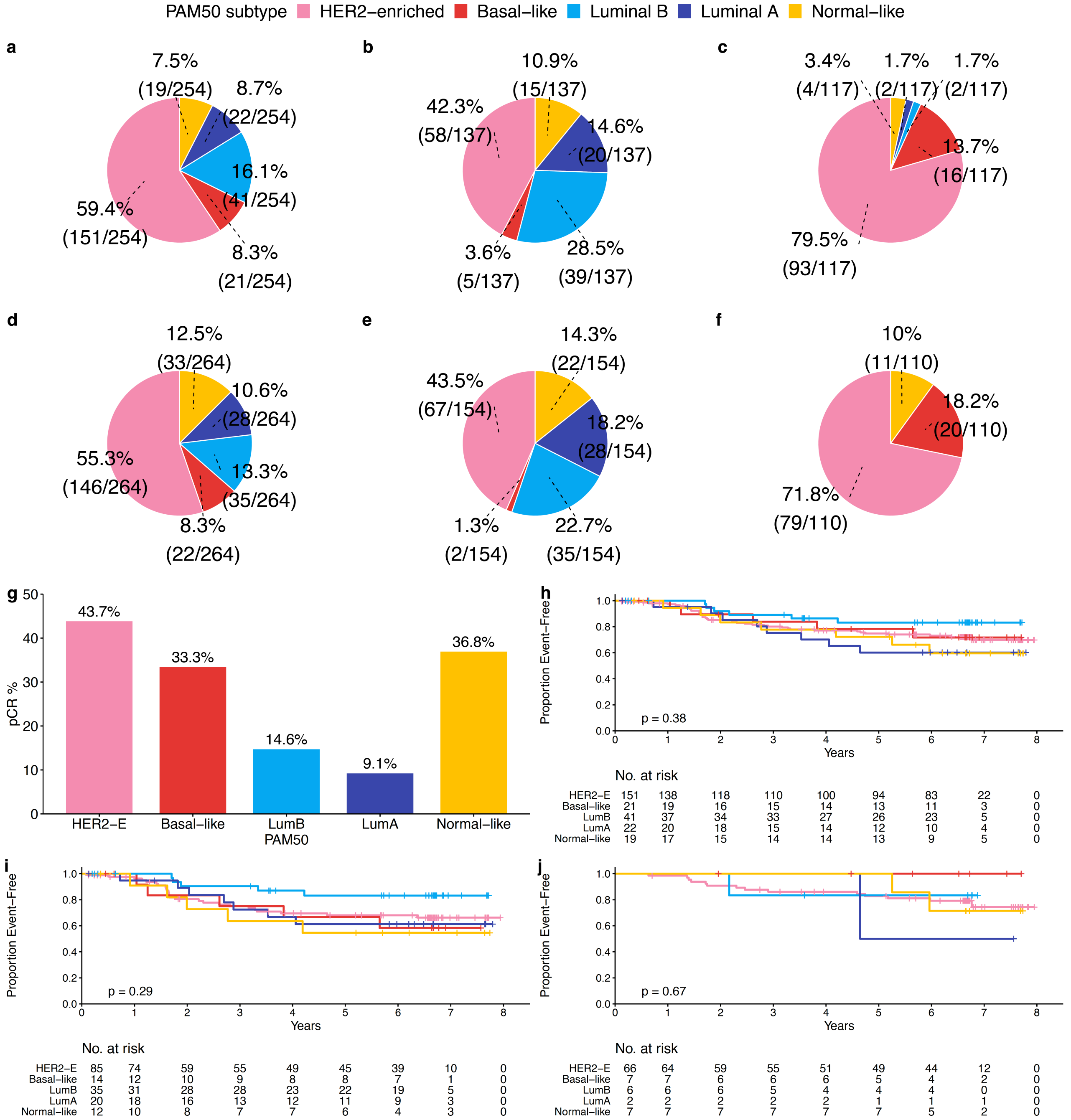

PAM50 subtype distribution in NeoALTTO and CALGB 40601, pCR rates and EFS outcomes according to PAM50 subtypes in NeoALTTO.

a Proportions of PAM50 subtypes in the whole NeoALTTO cohort (N = 254). **b** Proportions of PAM50 subtypes in the subgroup with hormone receptor-positive tumors from the NeoALTTO cohort (N = 137). **c** Proportions of PAM50 subtypes in the subgroup with hormone receptor-negative tumors from the NeoALTTO cohort (N = 117). **d** Proportions of PAM50 subtypes in the whole CALGB 40601 cohort (N = 264). **e** Proportions of PAM50 subtypes in the subgroup with hormone receptor-positive tumors from the CALGB 40601 cohort (N = 154). **f** Proportions of PAM50 subtypes in the subgroup with hormone receptor-negative tumors from the CALGB 40601 cohort (N = 110). **g** pCR (ypT0/is) rates in PAM50 subtypes in NeoALTTO. **h** Kaplan–Meier plot showing EFS according to PAM50 subtypes in the whole NeoALTTO cohort (N = 254). **i** Kaplan–Meier plot showing EFS according to PAM50 subtypes in the subgroup without breast pCR from the NeoALTTO cohort (N = 166). **j** Kaplan–Meier plot showing EFS according to PAM50 subtypes in the subgroup with breast pCR from the NeoALTTO cohort (N = 88). PAM50 groups available

in Supplementary data 9. P values in Kaplan-Meier plots are from log-rank test. The color legend for all plots is shown at the top of the figure.

EFS: event-free survival; HER2-E: HER2-Enriched; LumA: luminal A; LumB: luminal B; pCR: pathological complete response.

Supplementary Figure 10

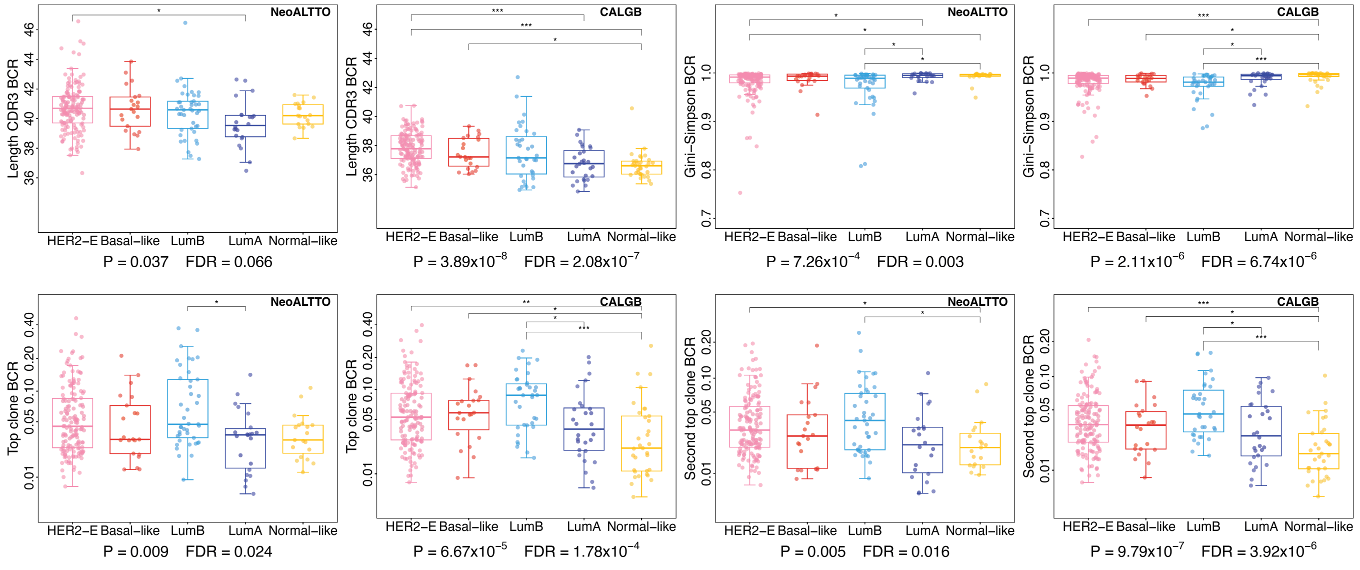

Additional BCR measures according to PAM50 subtypes.

Comparisons of BCR length of CDR3 (“Length CDR3”), Gini-Simpson index, top clone proportion (represented on a log scale) and second top clone proportion (represented on a log scale) in PAM50 subtypes in HER2-positive breast cancer in NeoALTTO and CALGB 40601. Statistical differences across groups were assessed using Kruskal-Wallis test (P values at the bottom of the panels) and Wilcoxon rank sum test (when comparing one group against each one of the others). FDRs were then obtained adjusting P values using Benjamini & Hochberg method (applied on the comparisons performed for all BCR measures in each study, separately). For Wilcoxon tests, FDRs < 0.05 are shown. In the panels: * = FDR < 0.05 and ≥ 0.01; ** = FDR < 0.01 and ≥ 0.001; *** = FDR < 0.001. P values are two-sided. See also Figure 1 for the other BCR measures. P values and FDRs available in Supplementary data 10 and 11. Source data are available.

In boxplots, the boxes are defined by the upper and lower quartile; the median is shown as a bold colored horizontal line; whiskers extend to the most extreme data point which is no more than 1.5 times the interquartile range from the box.

BCR: B cell receptor; CDR3: complementarity-determining region 3; FDR: false discovery rate; HER2-E: HER2-Enriched; LumA: luminal A; LumB: luminal B.

Supplementary Figure 11

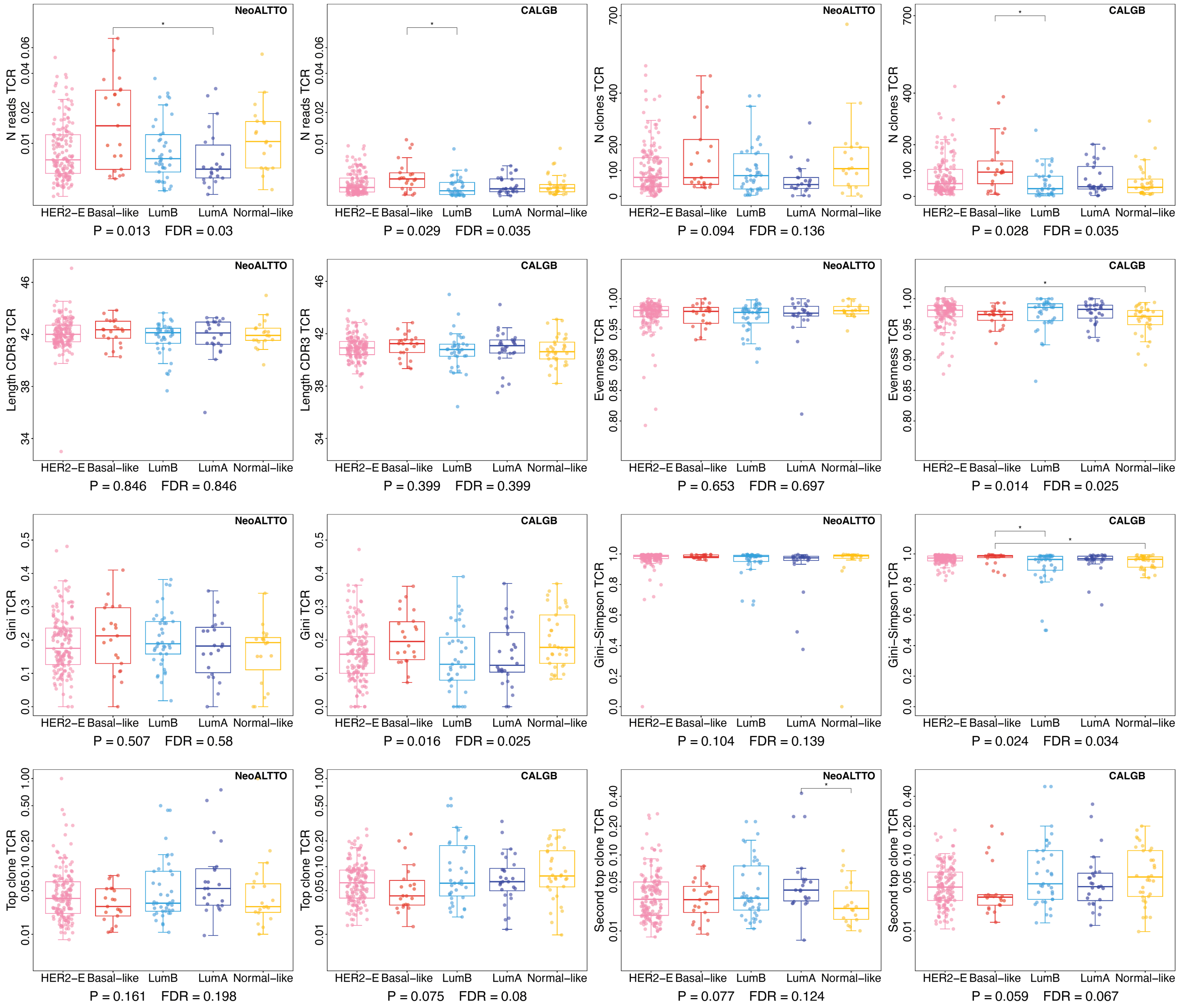

TCR measures according to PAM50 subtypes.

Comparisons of TCR normalized number of reads ("N reads"; represented on a log scale), number of clones ("N clones"), length of CDR3 ("Length CDR3 T"), evenness, Gini index, Gini-Simpson index, top clone proportion (represented on a log scale) and second top clone proportion (represented on a log scale) in PAM50 subtypes in HER2-positive breast cancer in NeoALTTO and CALGB 40601. Statistical differences across groups were assessed using Kruskal-Wallis test (P values at the bottom of the panels) and Wilcoxon rank sum test (when comparing one group against each one of the others). FDRs were then obtained adjusting P values using Benjamini & Hochberg method (applied on the comparisons performed for all BCR measures in each study, separately). For Wilcoxon tests, FDRs < 0.05 are shown. In the panels: * = FDR < 0.05 and ≥ 0.01; ** = FDR < 0.01 and ≥ 0.001; *** = FDR < 0.001. P values are two-sided. P values and FDRs available in Supplementary data 10 and 11. Source data are available. The number of reads is normalized by the total number of reads mapping to the transcriptome in each sample, and multiplied by 1000.

In boxplots, the boxes are defined by the upper and lower quartile; the median is shown as a bold colored horizontal line; whiskers extend to the most extreme data point which is no more than 1.5 times the interquartile range from the box.

BCR: B cell receptor; CDR3: complementarity-determining region 3; FDR: false discovery rate; HER2-E: HER2-Enriched; LumA: luminal A; LumB: luminal B; TCR: T cell receptor.

Supplementary Figure 12

a NeoALTTO

	OR	95% CI	*p*	FDR
BCR N reads	1.2	0.92 to 1.6	0.19	0.38
BCR N clones	1.2	0.88 to 1.5	0.29	0.46
BCR CDR3 length	0.97	0.75 to 1.3	0.8	0.8
BCR evenness	0.86	0.67 to 1.1	0.27	0.46
BCR Gini	1.2	0.92 to 1.6	0.18	0.38
BCR Gini–Simpson	0.96	0.74 to 1.2	0.74	0.8
BCR top clone	1.1	0.86 to 1.4	0.4	0.49
BCR second top clone	1.1	0.86 to 1.5	0.39	0.49
TCR N reads	1.2	0.94 to 1.6	0.13	0.34
TCR N clones	1.4	1.1 to 1.9	0.016	0.066
TCR CDR3 length	1.1	0.86 to 1.5	0.36	0.49
TCR evenness	1.8	1.1 to 2.7	0.012	0.065
TCR Gini	0.77	0.59 to 1	0.057	0.18
TCR Gini–Simpson	1.0	0.79 to 1.4	0.77	0.8
TCR top clone	0.6	0.44 to 0.82	0.0013	0.011
TCR second top clone	0.55	0.4 to 0.75	$1.4x10^{-4}$	0.0023

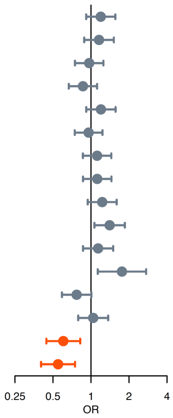

b CALGB 40601

	OR	95% CI	*p*	FDR
BCR N reads	2.0	1.5 to 2.7	$1.8x10^{-6}$	$2.3x10^{-5}$
BCR N clones	1.8	1.3 to 2.4	$5.7x10^{-5}$	$3.0x10^{-4}$
BCR CDR3 length	1.7	1.3 to 2.2	$1.7x10^{-4}$	$6.8x10^{-4}$
BCR evenness	0.63	0.48 to 0.82	$4.7x10^{-4}$	0.0015
BCR Gini	1.9	1.5 to 2.5	$2.9x10^{-6}$	$2.3x10^{-5}$
BCR Gini–Simpson	1.0	0.78 to 1.3	0.99	0.99
BCR top clone	1.0	0.81 to 1.3	0.8	0.92
BCR second top clone	0.99	0.77 to 1.3	0.92	0.98
TCR N reads	1.4	1.1 to 1.8	0.015	0.024
TCR N clones	1.4	1.1 to 1.8	0.0064	0.011
TCR CDR3 length	1.1	0.87 to 1.4	0.41	0.55
TCR evenness	1.1	0.88 to 1.5	0.32	0.47
TCR Gini	0.97	0.76 to 1.2	0.78	0.92
TCR Gini–Simpson	2.0	1.3 to 3	0.0029	0.0067
TCR top clone	0.66	0.51 to 0.85	0.0016	0.0043
TCR second top clone	0.7	0.54 to 0.9	0.0056	0.011

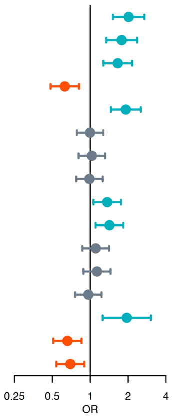

c NeoALTTO

	OR	95% CI	*p*	FDR
BCR N reads	1.1	0.83 to 1.5	0.48	0.79
BCR N clones	1.1	0.84 to 1.5	0.44	0.79
BCR CDR3 length	0.88	0.66 to 1.2	0.39	0.79
BCR evenness	0.95	0.72 to 1.3	0.75	0.86
BCR Gini	1.1	0.81 to 1.4	0.6	0.86
BCR Gini–Simpson	1.0	0.76 to 1.3	0.99	1.0
BCR top clone	1.1	0.8 to 1.4	0.67	0.86
BCR second top clone	1.1	0.8 to 1.4	0.7	0.86
TCR N reads	1.2	0.9 to 1.6	0.21	0.57
TCR N clones	1.4	1 to 1.8	0.035	0.11
TCR CDR3 length	1.1	0.83 to 1.5	0.49	0.79
TCR evenness	2.0	1.3 to 3.3	$4.2x10^{-4}$	0.0034
TCR Gini	0.68	0.51 to 0.92	0.011	0.042
TCR Gini–Simpson	1.0	0.76 to 1.3	1.0	1.0
TCR top clone	0.6	0.43 to 0.84	0.0015	0.008
TCR second top clone	0.56	0.4 to 0.77	$2.1x10^{-4}$	0.0034

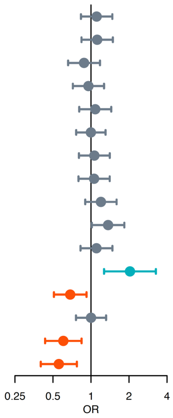

d CALGB 40601

	OR	95% CI	*p*	FDR
BCR N reads	1.8	1.3 to 2.6	$2.3x10^{-4}$	0.0036
BCR N clones	1.7	1.2 to 2.4	0.001	0.0084
BCR CDR3 length	1.4	1.1 to 1.9	0.014	0.057
BCR evenness	0.73	0.54 to 0.98	0.037	0.098
BCR Gini	1.6	1.2 to 2.2	0.0039	0.021
BCR Gini–Simpson	1.0	0.76 to 1.3	1.0	1.0
BCR top clone	0.91	0.68 to 1.2	0.51	0.68
BCR second top clone	0.86	0.64 to 1.1	0.31	0.45
TCR N reads	1.3	0.96 to 1.8	0.084	0.13
TCR N clones	1.3	0.99 to 1.8	0.056	0.11
TCR CDR3 length	1.0	0.8 to 1.4	0.74	0.85
TCR evenness	1.1	0.81 to 1.4	0.68	0.84
TCR Gini	0.99	0.75 to 1.3	0.93	0.99
TCR Gini–Simpson	1.7	1 to 2.8	0.02	0.063
TCR top clone	0.74	0.55 to 1	0.045	0.1
TCR second top clone	0.76	0.56 to 1	0.068	0.12

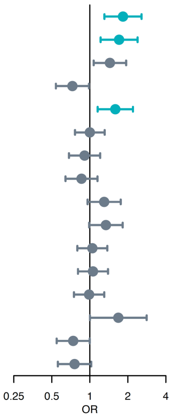

Association of BCR and TCR measures with pCR in the breast (ypT0/is) in the NeoALTTO and CALGB 40601 cohorts.

a Forest plot for pCR (ypT0/is) in the NeoALTTO cohort, univariable analysis. **b** Forest plot for pCR in the CALGB 40601 cohort, univariable analysis. **c** Forest plot for pCR in the NeoALTTO cohort, correcting for clinicopathological parameters (age, hormone receptor status, tumor size, nodal status, PAM50 subtypes, and treatment arm). **d** Forest plot for pCR in the CALGB 40601 cohort, correcting for clinicopathological parameters (age, hormone receptor status, tumor size, nodal status, PAM50 subtypes, and treatment arm).

For univariable analysis, P values are from logistic regression. When correcting for clinicopathological characteristics, P values were obtained with an ANOVA on nested logistic models. P values are two-sided.

Non-significant values (FDR > 0.05) are shown in dark grey, significant values are shown in red (OR < 1) and blue (OR > 1). Circles indicate odds ratio (OR), and error bars the 95% confidence interval (95% CI). Analyses were performed including patients with available data. In NeoALTTO, N = 254 for all BCR/TCR metrics, except TCR CDR3 length, TCR Gini, TCR Gini-Simpson, TCR top clone (N = 253) and TCR evenness, TCR second top clone (N = 251). In CALGB 40601, for all BCR/TCR measures N = 264 in univariable, N = 248 in multivariable.

BCR: B cell receptor; CDR3: complementarity-determining region 3; CI: 95% confidence interval; FDR: false discovery rate; N reads: number of normalized reads; N clones: number of clones; OR: odds ratio; pCR: pathological complete response; TCR: T cell receptor.

Supplementary Figure 13

a NeoALTTO

	OR	95% CI	*p*	FDR
BCR N reads	1.1	0.83 to 1.4	0.53	0.71
BCR N clones	1.1	0.8 to 1.4	0.71	0.76
BCR CDR3 length	0.91	0.69 to 1.2	0.49	0.71
BCR evenness	0.93	0.71 to 1.2	0.58	0.71
BCR Gini	1.1	0.81 to 1.4	0.62	0.71
BCR Gini–Simpson	0.93	0.72 to 1.2	0.57	0.71
BCR top clone	1.1	0.87 to 1.5	0.35	0.65
BCR second top clone	1.2	0.88 to 1.5	0.31	0.65
TCR N reads	1.2	0.9 to 1.6	0.22	0.58
TCR N clones	1.3	0.96 to 1.7	0.096	0.39
TCR CDR3 length	1.1	0.86 to 1.5	0.37	0.65
TCR evenness	1.5	1 to 2.3	0.052	0.28
TCR Gini	0.82	0.62 to 1.1	0.17	0.55
TCR Gini–Simpson	0.99	0.76 to 1.3	0.91	0.91
TCR top clone	0.69	0.51 to 0.93	0.017	0.13
TCR second top clone	0.63	0.46 to 0.86	0.0035	0.056

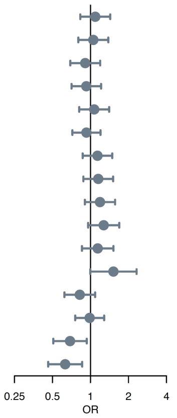

b CALGB 40601

	OR	95% CI	*p*	FDR
BCR N reads	1.8	1.4 to 2.4	3.2×10^{-5}	2.6×10^{-4}
BCR N clones	1.6	1.2 to 2.2	4.9×10^{-4}	0.0025
BCR CDR3 length	1.5	1.2 to 2	0.0013	0.0042
BCR evenness	0.64	0.49 to 0.82	6.1×10^{-4}	0.0025
BCR Gini	1.8	1.4 to 2.4	2.2×10^{-5}	2.6×10^{-4}
BCR Gini–Simpson	0.95	0.75 to 1.2	0.7	0.81
BCR top clone	1.0	0.81 to 1.3	0.79	0.81
BCR second top clone	1.0	0.81 to 1.3	0.81	0.81
TCR N reads	1.3	0.98 to 1.6	0.075	0.12
TCR N clones	1.3	1 to 1.7	0.029	0.051
TCR CDR3 length	1.1	0.86 to 1.4	0.46	0.61
TCR evenness	1.1	0.88 to 1.5	0.32	0.47
TCR Gini	0.97	0.76 to 1.2	0.79	0.81
TCR Gini–Simpson	1.7	1.1 to 2.7	0.01	0.024
TCR top clone	0.71	0.55 to 0.92	0.0091	0.024
TCR second top clone	0.75	0.58 to 0.96	0.025	0.05

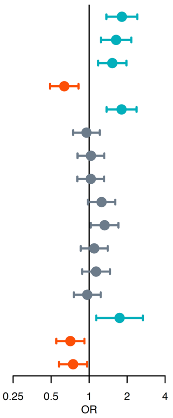

c NeoALTTO

	OR	95% CI	*p*	FDR
BCR N reads	0.99	0.74 to 1.3	0.97	0.97
BCR N clones	1.0	0.76 to 1.4	0.93	0.97
BCR CDR3 length	0.81	0.6 to 1.1	0.18	0.53
BCR evenness	1.0	0.77 to 1.4	0.83	0.94
BCR Gini	0.95	0.7 to 1.3	0.73	0.94
BCR Gini–Simpson	0.96	0.74 to 1.3	0.79	0.94
BCR top clone	1.1	0.8 to 1.4	0.62	0.94
BCR second top clone	1.1	0.81 to 1.5	0.55	0.94
TCR N reads	1.1	0.84 to 1.5	0.43	0.94
TCR N clones	1.2	0.9 to 1.6	0.2	0.53
TCR CDR3 length	1.1	0.81 to 1.5	0.55	0.94
TCR evenness	1.8	1.1 to 2.9	0.0033	0.051
TCR Gini	0.72	0.53 to 0.98	0.031	0.13
TCR Gini–Simpson	0.95	0.73 to 1.2	0.73	0.94
TCR top clone	0.69	0.5 to 0.96	0.021	0.11
TCR second top clone	0.64	0.46 to 0.89	0.0064	0.051

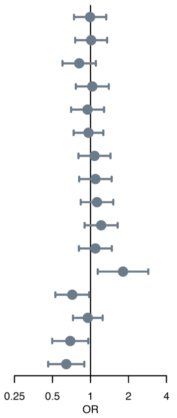

d CALGB 40601

	OR	95% CI	*p*	FDR
BCR N reads	1.7	1.2 to 2.4	0.0015	0.025
BCR N clones	1.6	1.1 to 2.3	0.0038	0.03
BCR CDR3 length	1.3	0.98 to 1.8	0.072	0.17
BCR evenness	0.74	0.55 to 1	0.047	0.16
BCR Gini	1.5	1.1 to 2	0.016	0.088
BCR Gini–Simpson	0.97	0.74 to 1.3	0.85	0.9
BCR top clone	0.9	0.68 to 1.2	0.48	0.63
BCR second top clone	0.89	0.66 to 1.2	0.44	0.63
TCR N reads	1.2	0.9 to 1.7	0.19	0.3
TCR N clones	1.3	0.94 to 1.7	0.11	0.2
TCR CDR3 length	1.0	0.79 to 1.4	0.78	0.89
TCR evenness	1.1	0.81 to 1.4	0.6	0.74
TCR Gini	0.99	0.75 to 1.3	0.94	0.94
TCR Gini–Simpson	1.5	0.93 to 2.6	0.049	0.16
TCR top clone	0.76	0.56 to 1	0.072	0.17
TCR second top clone	0.78	0.57 to 1.1	0.11	0.2

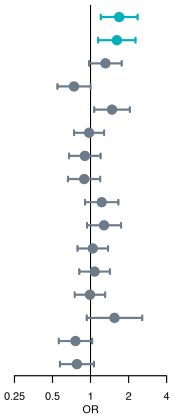

Association of BCR and TCR measures with pCR in the breast + axilla (ypT0/is ypN0) in the NeoALTTO and CALGB 40601 cohorts.

a Forest plot for pCR (ypT0/is ypN0) in the NeoALTTO cohort, univariable analysis. **b** Forest plot for pCR in the CALGB 40601 cohort, univariable analysis. **c** Forest plot for pCR in the NeoALTTO cohort, correcting for clinicopathological parameters (age, hormone receptor status, tumor size, nodal status, PAM50 subtypes, and treatment arm). **d** Forest plot for pCR in the CALGB 40601 cohort, correcting for clinicopathological parameters (age, hormone receptor status, tumor size, nodal status, PAM50 subtypes, and treatment arm).

For univariable analysis, P values are from logistic regression. When correcting for clinicopathological characteristics, P values were obtained with an ANOVA on nested logistic models. P values are two-sided.

Non-significant values (FDR > 0.05) are shown in dark grey, significant values are shown in red (OR < 1) and blue (OR > 1). Circles indicate OR, and error bars the 95% confidence interval (95% CI). Analyses were performed including patients with available data. In NeoALTTO, N = 244 for all BCR/TCR metrics, except TCR CDR3 length, TCR Gini, TCR Gini-Simpson, TCR top clone (N = 243) and TCR evenness, TCR second top clone (N = 241). In CALGB 40601, for all BCR/TCR measures N = 264 in univariable, N = 248 in multivariable.

BCR: B cell receptor; CDR3: complementarity-determining region 3; CI: 95% confidence interval; FDR: false discovery rate; N reads: number of normalized reads; N clones: number of clones; OR: odds ratio; pCR: pathological complete response; TCR: T cell receptor.

Supplementary Figure 14

a NeoALTTO

	HR	95% CI	*p*	FDR
IGH N reads	0.76	0.59 to 0.97	0.031	0.11
IGK N reads	0.74	0.58 to 0.96	0.023	0.093
IGL N reads	0.8	0.63 to 1	0.078	0.19
IGH N clones	0.86	0.69 to 1.1	0.22	0.37
IGK N clones	0.92	0.71 to 1.2	0.52	0.7
IGL N clones	0.86	0.68 to 1.1	0.23	0.37
IGH CDR3 length	0.91	0.71 to 1.2	0.44	0.63
IGK CDR3 length	0.81	0.6 to 1.1	0.12	0.25
IGL CDR3 length	1.1	0.83 to 1.4	0.6	0.75
IGH evenness	1.4	1 to 1.8	0.017	0.08
IGK evenness	1.6	1.2 to 2.1	0.0015	0.018
IGL evenness	1.3	0.97 to 1.7	0.068	0.18
IGH Gini	0.75	0.6 to 0.93	0.012	0.075
IGK Gini	0.67	0.53 to 0.85	0.0012	0.018
IGL Gini	0.72	0.56 to 0.91	0.0077	0.062
IGH Gini–Simpson	1.3	0.8 to 2.3	0.15	0.28
IGK Gini–Simpson	1.3	0.9 to 1.9	0.11	0.24
IGL Gini–Simpson	1.0	0.78 to 1.4	0.82	0.94
IGH top clone	1.0	0.79 to 1.3	0.93	0.99
IGK top clone	0.88	0.68 to 1.1	0.31	0.46
IGL top clone	1.0	0.77 to 1.3	0.99	0.99
IGH second top clone	0.95	0.73 to 1.2	0.69	0.83
IGK second top clone	0.79	0.61 to 1	0.068	0.18
IGL second top clone	1.0	0.78 to 1.3	0.94	0.99

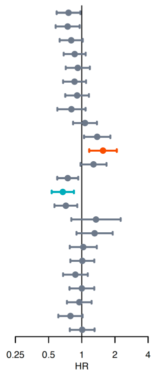

b CALGB 40601

	HR	95% CI	*p*	FDR
IGH N reads	0.5	0.36 to 0.68	$1.3x10^{-5}$	$1.5x10^{-4}$
IGK N reads	0.51	0.38 to 0.69	$1.5x10^{-5}$	$1.5x10^{-4}$
IGL N reads	0.52	0.39 to 0.7	$1.9x10^{-5}$	$1.5x10^{-4}$
IGH N clones	0.55	0.41 to 0.74	$8.8x10^{-5}$	$5.3x10^{-4}$
IGK N clones	0.6	0.46 to 0.79	$3.8x10^{-4}$	0.0015
IGL N clones	0.57	0.43 to 0.75	$1.3x10^{-4}$	$6.4x10^{-4}$
IGH CDR3 length	0.96	0.72 to 1.3	0.81	0.81
IGK CDR3 length	1.0	0.77 to 1.4	0.79	0.81
IGL CDR3 length	1.0	0.79 to 1.4	0.75	0.81
IGH evenness	1.3	0.89 to 1.8	0.17	0.28
IGK evenness	1.7	1.2 to 2.4	0.0041	0.012
IGL evenness	1.5	1 to 2.2	0.021	0.039
IGH Gini	0.66	0.47 to 0.92	0.014	0.034
IGK Gini	0.6	0.43 to 0.82	0.0019	0.0066
IGL Gini	0.64	0.47 to 0.87	0.0054	0.014
IGH Gini–Simpson	0.92	0.77 to 1.1	0.42	0.56
IGK Gini–Simpson	1.3	0.87 to 1.9	0.18	0.28
IGL Gini–Simpson	1.1	0.74 to 1.5	0.72	0.81
IGH top clone	1.4	1.1 to 1.9	0.018	0.039
IGK top clone	1.1	0.79 to 1.4	0.66	0.79
IGL top clone	1.2	0.85 to 1.6	0.34	0.48
IGH second top clone	1.5	1.1 to 2	0.02	0.039
IGK second top clone	1.2	0.9 to 1.7	0.19	0.29
IGL second top clone	1.1	0.82 to 1.6	0.44	0.56

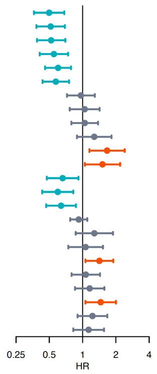

c NeoALTTO

	HR	95% CI	*p*	FDR
IgG N reads	0.72	0.56 to 0.92	0.01	0.084
IgG N clones	0.76	0.61 to 0.95	0.024	0.14
IgG CDR3 length	0.96	0.75 to 1.2	0.73	0.77
IgG evenness	1.2	0.92 to 1.6	0.15	0.26
IgG Gini	0.81	0.65 to 1	0.085	0.25
IgG Gini–Simpson	1.1	0.81 to 1.5	0.54	0.64
IgG top clone	1.1	0.91 to 1.5	0.26	0.34
IgG second top clone	1.3	0.97 to 1.6	0.079	0.25
IgM N reads	0.78	0.62 to 1	0.048	0.23
IgM N clones	0.83	0.66 to 1	0.12	0.25
IgM CDR3 length	1.2	0.91 to 1.5	0.23	0.32
IgM evenness	1.2	0.93 to 1.7	0.12	0.25
IgM Gini	0.69	0.53 to 0.89	0.0042	0.063
IgM Gini–Simpson	0.95	0.74 to 1.2	0.7	0.77
IgM top clone	0.94	0.73 to 1.2	0.62	0.71
IgM second top clone	0.84	0.66 to 1.1	0.16	0.26
IgA N reads	1.2	0.9 to 1.6	0.21	0.31
IgA N clones	1.2	0.87 to 1.5	0.31	0.39
IgA CDR3 length	0.69	0.54 to 0.88	0.0052	0.063
IgA evenness	1.3	0.93 to 1.7	0.11	0.25
IgA Gini	0.82	0.63 to 1.1	0.13	0.25
IgA Gini–Simpson	1.0	0.74 to 1.4	0.91	0.91
IgA top clone	0.81	0.62 to 1	0.1	0.25
IgA second top clone	0.82	0.63 to 1.1	0.15	0.26

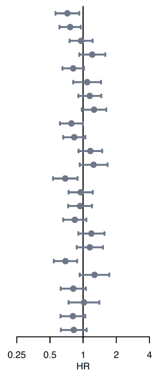

d CALGB 40601

	HR	95% CI	*p*	FDR
IgG N reads	0.53	0.38 to 0.74	$1.7x10^{-4}$	0.0022
IgG N clones	0.57	0.42 to 0.77	$4.1x10^{-4}$	0.0025
IgG CDR3 length	0.92	0.69 to 1.2	0.59	0.62
IgG evenness	1.1	0.78 to 1.5	0.63	0.63
IgG Gini	0.67	0.49 to 0.9	0.011	0.026
IgG Gini–Simpson	0.77	0.65 to 0.91	0.01	0.026
IgG top clone	1.5	1.1 to 2	0.0042	0.012
IgG second top clone	1.3	1 to 1.8	0.048	0.08
IgM N reads	0.56	0.41 to 0.76	$2.0x10^{-4}$	0.0022
IgM N clones	0.63	0.47 to 0.84	0.0019	0.0084
IgM CDR3 length	1.2	0.83 to 1.7	0.35	0.38
IgM evenness	1.4	0.94 to 2	0.074	0.11
IgM Gini	0.66	0.47 to 0.91	0.012	0.027
IgM Gini–Simpson	0.85	0.64 to 1.1	0.29	0.33
IgM top clone	1.3	0.91 to 1.7	0.16	0.2
IgM second top clone	1.4	1 to 1.9	0.038	0.069
IgA N reads	0.61	0.47 to 0.78	$2.7x10^{-4}$	0.0022
IgA N clones	0.65	0.5 to 0.84	0.0021	0.0084
IgA CDR3 length	0.78	0.56 to 1.1	0.13	0.19
IgA evenness	1.3	0.88 to 2	0.15	0.19
IgA Gini	0.61	0.44 to 0.85	0.0031	0.01
IgA Gini–Simpson	0.75	0.6 to 0.92	0.017	0.034
IgA top clone	1.4	1 to 2	0.05	0.08
IgA second top clone	1.3	0.92 to 1.8	0.14	0.19

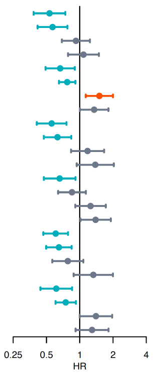

Association of BCR chains and immunoglobulin isotypes with EFS at multivariable analysis in the NeoALTTO and CALGB 40601 cohorts.

a BCR heavy (IGH) and light (IGK, IGL) chains, forest plot for EFS in the NeoALTTO cohort, multivariable analysis. **b** BCR heavy (IGH) and light (IGK, IGL) chains, forest plot for EFS in the CALGB 40601 cohort, multivariable analysis.

c Immunoglobulin isotypes (IgG, IgM, IgA), forest plot for EFS in the NeoALTTO cohort, multivariable analysis. **d** Immunoglobulin isotypes (IgG, IgM, IgA), forest plot for EFS in the CALGB 40601 cohort, multivariable analysis.

The analysis on isotypes was focused on IgG, IgM and IgA due to the low proportions of IgD and IgE detected. All analyses were corrected for clinicopathological parameters (age, hormone receptor status, tumor size, nodal status, PAM50 subtypes, and treatment arm). P values were obtained with an ANOVA on nested Cox models. P values are two-sided.

Non-significant values (FDR > 0.05) are shown in dark grey, significant values are shown in red (HR > 1) and blue (HR <1). Circles indicate HR, and error bars the 95% confidence interval (95% CI). Analyses were performed including patients with available data. For IGH/IGK/IGL in NeoALTTO, N = 254 for all IGH/IGK/IGL metrics, except IGL CDR3 length, IGL evenness, IGL Gini, IGL Gini-Simpson, IGL top clone, IGL second top clone (N = 253). For IGH/IGK/IGL in CALGB 40601, N = 248 for all IGH/IGK/IGL metrics, except IGH evenness, IGH second top clone (N = 247). For IgG/IgM/IgA in NeoALTTO, N = 254 for IgG/IgM/IgA N reads, IgG/IgM/IgA N clones, IgG CDR3 length, IgG Gini, IgG Gini-Simpson, IgG top clone; N = 253 for IgG evenness, IgG second top clone; N = 252 for IgA CDR3 length, IgA Gini, IgA Gini-Simpson, IgA top clone; N = 249 for IgA evenness, IgA second top clone; N = 247 for IgM CDR3 length, IgM Gini, IgM Gini-Simpson, IgM top clone; N = 243 for IgM evenness, IgM second top clone. For IgG/IgM/IgA in CALGB 40601, N = 248 for IgG/IgM/IgA N reads, IgG/IgM/IgA N clones; N = 246 for IgG CDR3 length, IgG Gini, IgG Gini-Simpson, IgG top clone; N = 245 for IgG evenness, IgG second top clone; N = 242 for IgA CDR3 length, IgA Gini, IgA Gini-Simpson, IgA top clone; N = 238 for IgA

evenness, IgA second top clone; N = 220 for IgM CDR3 length, IgM Gini, IgM Gini-Simpson, IgM top clone; N = 207 for IgM evenness, IgM second top clone.

95% CI: 95% confidence interval; BCR: B cell receptor; CDR3: complementarity-determining region 3; EFS: event-free survival; FDR: false discovery rate; HR: hazard ratio; Ig: immunoglobulin; N reads: number of normalized reads; N clones: number of clones.

Supplementary Figure 15

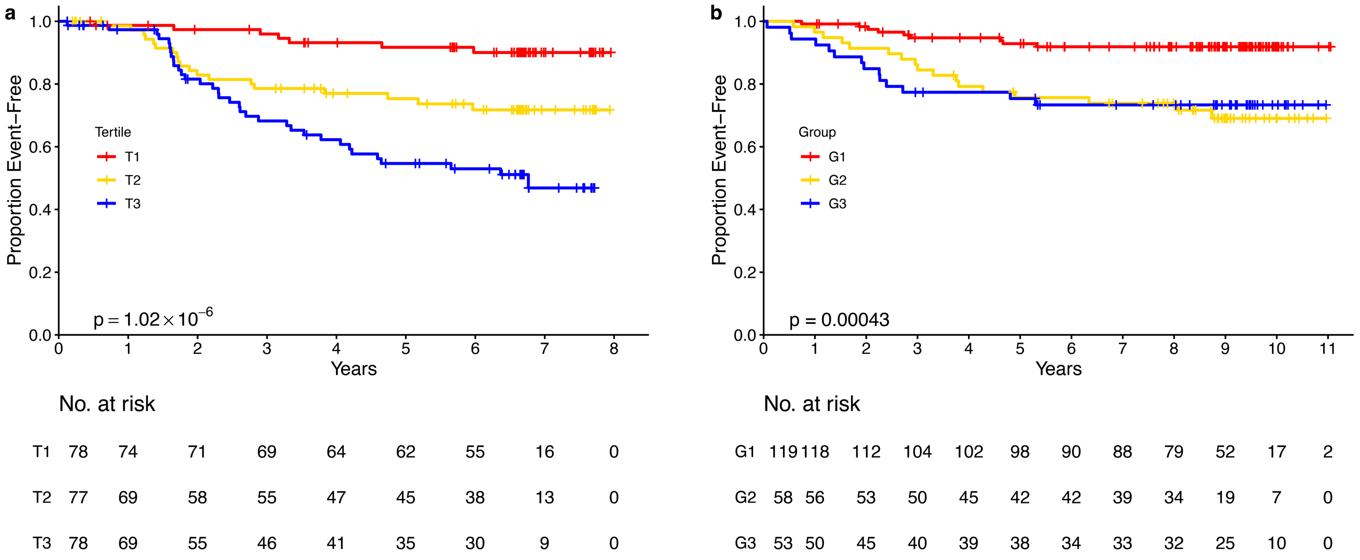

Event-free survival outcomes based on the tertiles identified in NeoALTTO, and in groups identified applying the same cutoffs in CALGB 40601.

a Kaplan–Meier plot showing EFS in the NeoALTTO population (N = 233) with information available for all variables included in the model (breast pCR, hormone receptor status, clinical nodal status, TILs, BCR evenness). Tertiles (first, second and third: T1, T2, T3, respectively) were identified based on the score derived from the prognostic model.

b Kaplan–Meier plot showing EFS in the CALGB 40601 population (N = 230) with information available for all variables included in the model. The cutoffs dividing tertiles in NeoALTTO (-1.3763 and -0.8143) were applied in the CALGB 40601, obtaining three groups (G1, G2, G3).

P values are from log-rank test.

BCR: B cell receptor; EFS: event-free survival; G: group; T: tertile; TILs: tumor-infiltrating lymphocytes.

Supplementary Figure 16

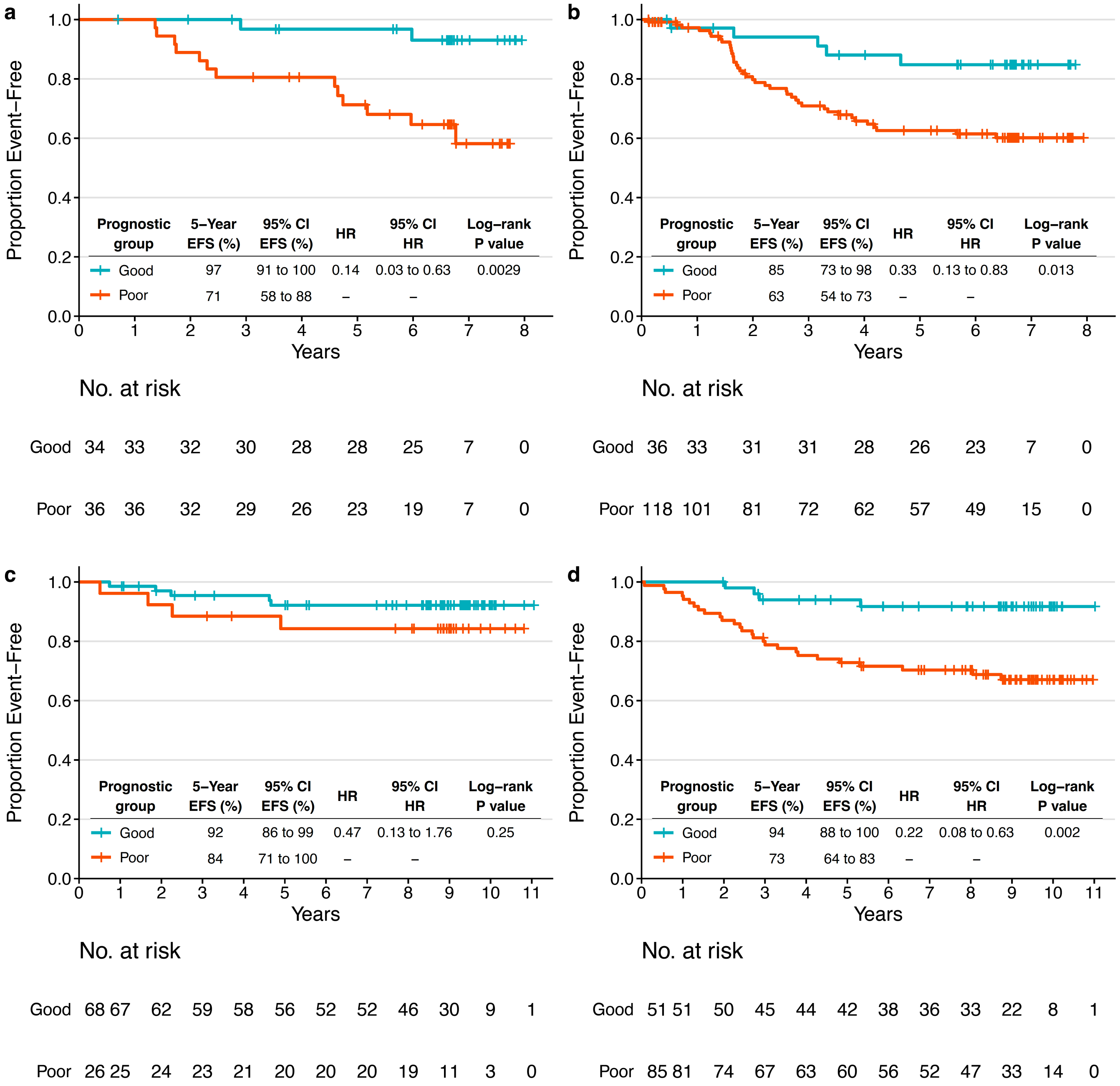

Event-free survival outcomes based on the groups identified by the prognostic model according to breast + axilla pCR definition (ypT0/is ypN0).

a Kaplan–Meier plot showing EFS in the NeoALTTO subgroup with all variables in the model available (breast pCR, hormone receptor status, clinical nodal status, TILs, BCR evenness) and breast + axilla pCR (ypT0/is ypN0) at surgery (N = 70). **b** Kaplan–Meier plot showing EFS in the NeoALTTO subgroup with all variables in the model available and with residual disease at surgery (N = 154).

c Kaplan–Meier plot showing EFS in the CALGB 40601 subgroup with all variables in the model available and breast + axilla pCR (ypT0/is ypN0) at surgery (N = 94). **d** Kaplan–Meier plot showing EFS in the CALGB 40601 subgroup with all variables in the model available and with residual disease at surgery (N = 136).

Patients are stratified according to low risk (good prognosis group) and high risk (poor prognosis), based on the score derived from the prognostic model. Patients with a score ≤ -1.3763 were assigned to the good prognosis group, based on the cutoff identified in the NeoALTTO cohort.

Tables show 5-year EFS rates and HRs with respective 95% CI. P values are from log-rank test, HR describes the risk of an event as defined by EFS in the good prognosis group compared to the one with poor prognosis.

95% CI: 95% confidence interval; BCR: B cell receptor; EFS: event-free survival; HR: hazard ratio; pCR: pathological complete response; TILs: tumor-infiltrating lymphocytes.

Supplementary Figure 17

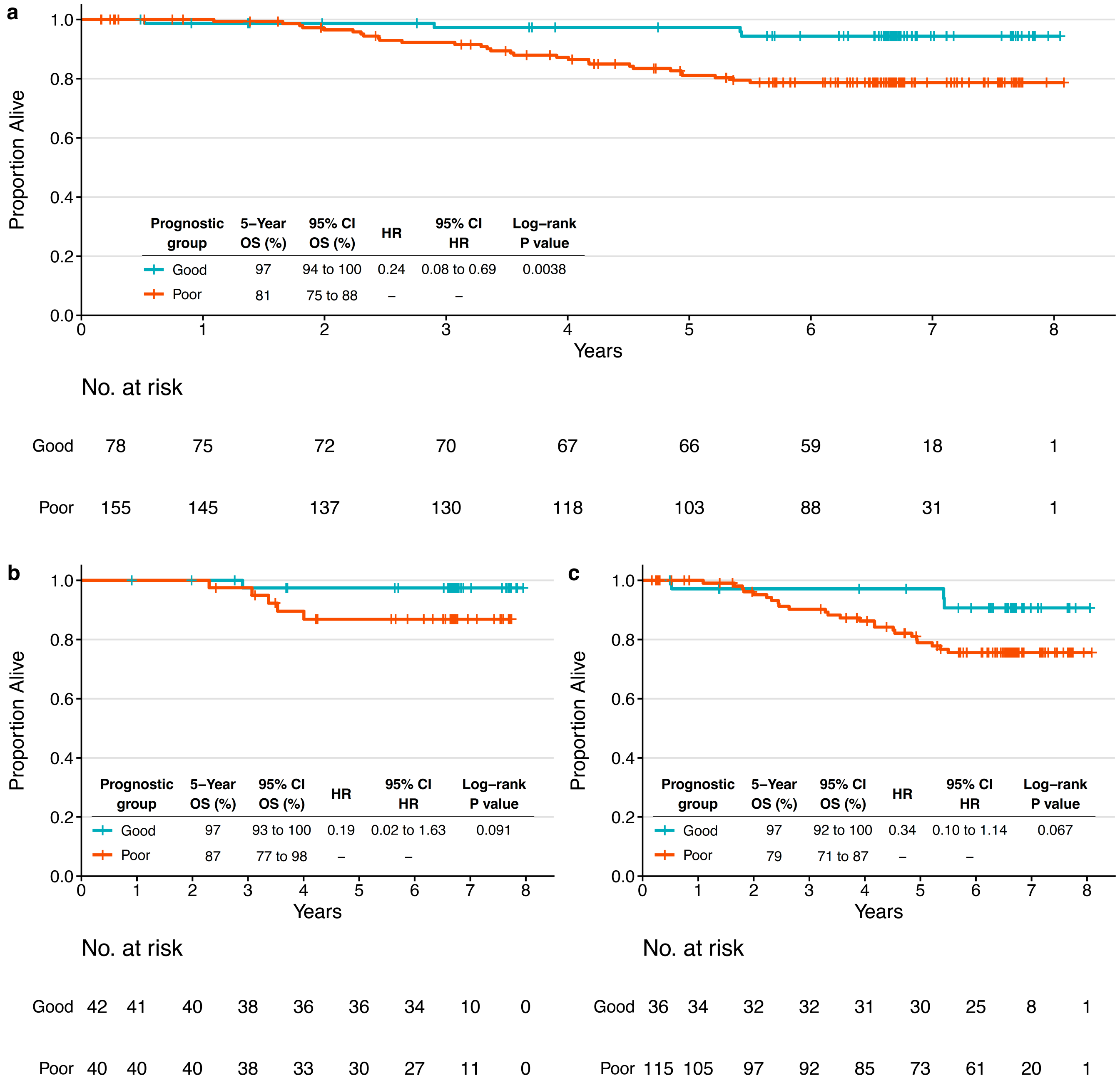

Overall survival outcomes based on the groups identified by the prognostic HER2-EveNT model in the NeoALTTO dataset.

a Kaplan–Meier plot showing OS in the NeoALTTO population (N = 233) with information available for all variables included in the model (breast pCR, hormone receptor status, clinical nodal status, TILs, BCR evenness). **b** Kaplan–Meier plot showing OS in the NeoALTTO subgroup with all variables in the model available and breast pCR (ypT0/is) at surgery (N = 82). **c** Kaplan–Meier plot showing OS in the NeoALTTO subgroup with all variables in the model available and without pCR in the breast at surgery (N = 151).

Tables show 5-year OS rates and HRs with respective 95% CI. P values are from log-rank test, HR describes the risk of an event as defined by OS in the good prognosis group compared to the one with poor prognosis.

95% CI: 95% confidence interval; BCR: B cell receptor; HR: hazard ratio; OS: overall survival; pCR: pathological complete response; TILs: tumor-infiltrating lymphocytes.

Supplementary Figure 18

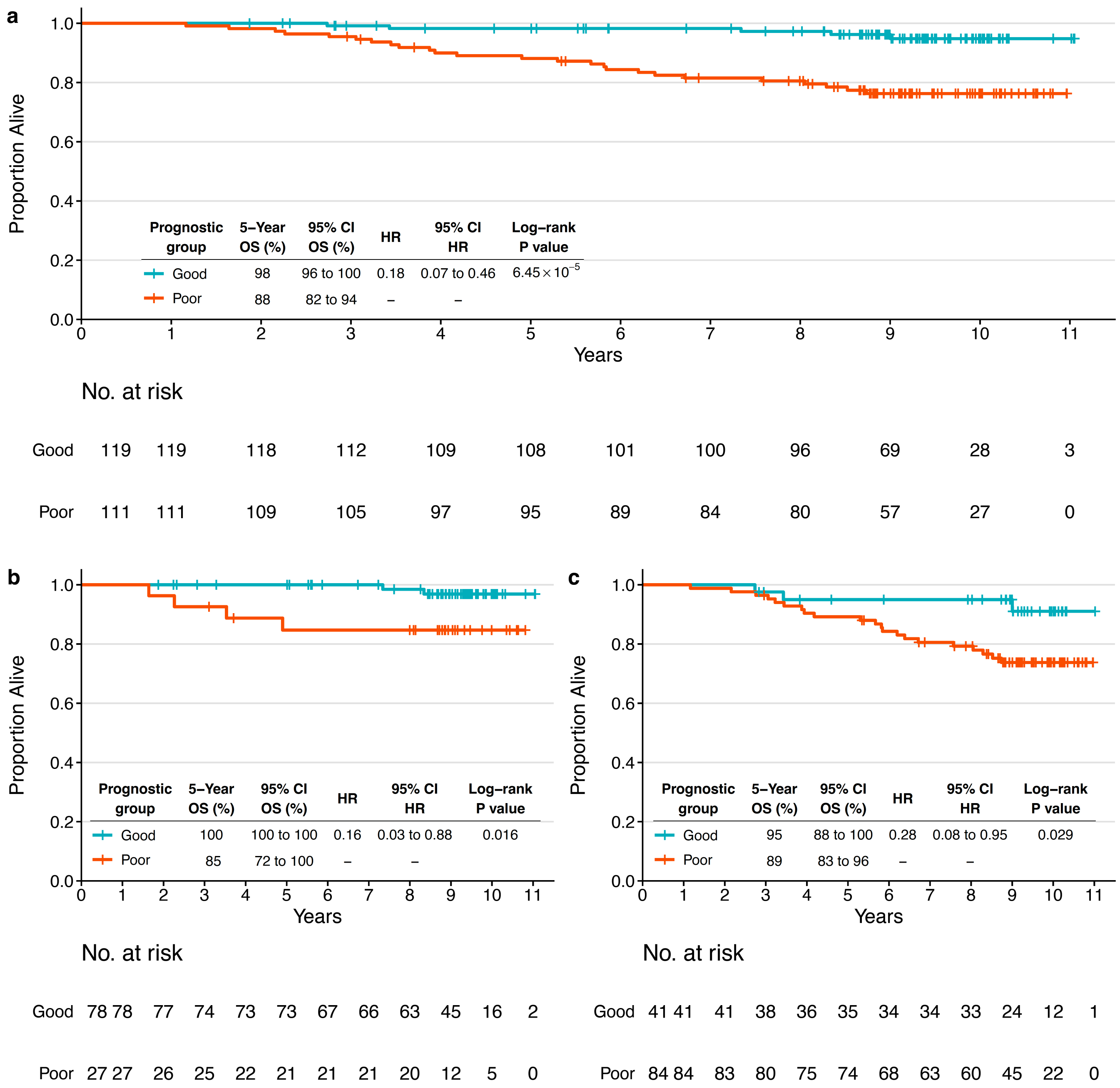

Overall survival outcomes based on the groups identified by the prognostic HER2-EveNT model in the CALGB 40601 independent validation dataset.

a Kaplan–Meier plot showing OS in the CALGB 40601 population (N = 230) with information available for all variables included in the model (breast pCR, hormone receptor status, clinical nodal status, TILs, BCR evenness). **b** Kaplan–Meier plot showing OS in the CALGB 40601 subgroup with all variables in the model available and breast pCR (ypT0/is) at surgery (N = 105). **c** Kaplan–Meier plot showing OS in the CALGB 40601 subgroup with all variables in the model available and without pCR in the breast at surgery (N = 125).

Tables show 5-year OS rates and HRs with respective 95% CI. P values are from log-rank test, HR describes the risk of an event as defined by OS in the good prognosis group compared to the one with poor prognosis.

95% CI: 95% confidence interval; BCR: B cell receptor; HR: hazard ratio; OS: overall survival; pCR: pathological complete response; TILs: tumor-infiltrating lymphocytes.

Supplementary Figure 19

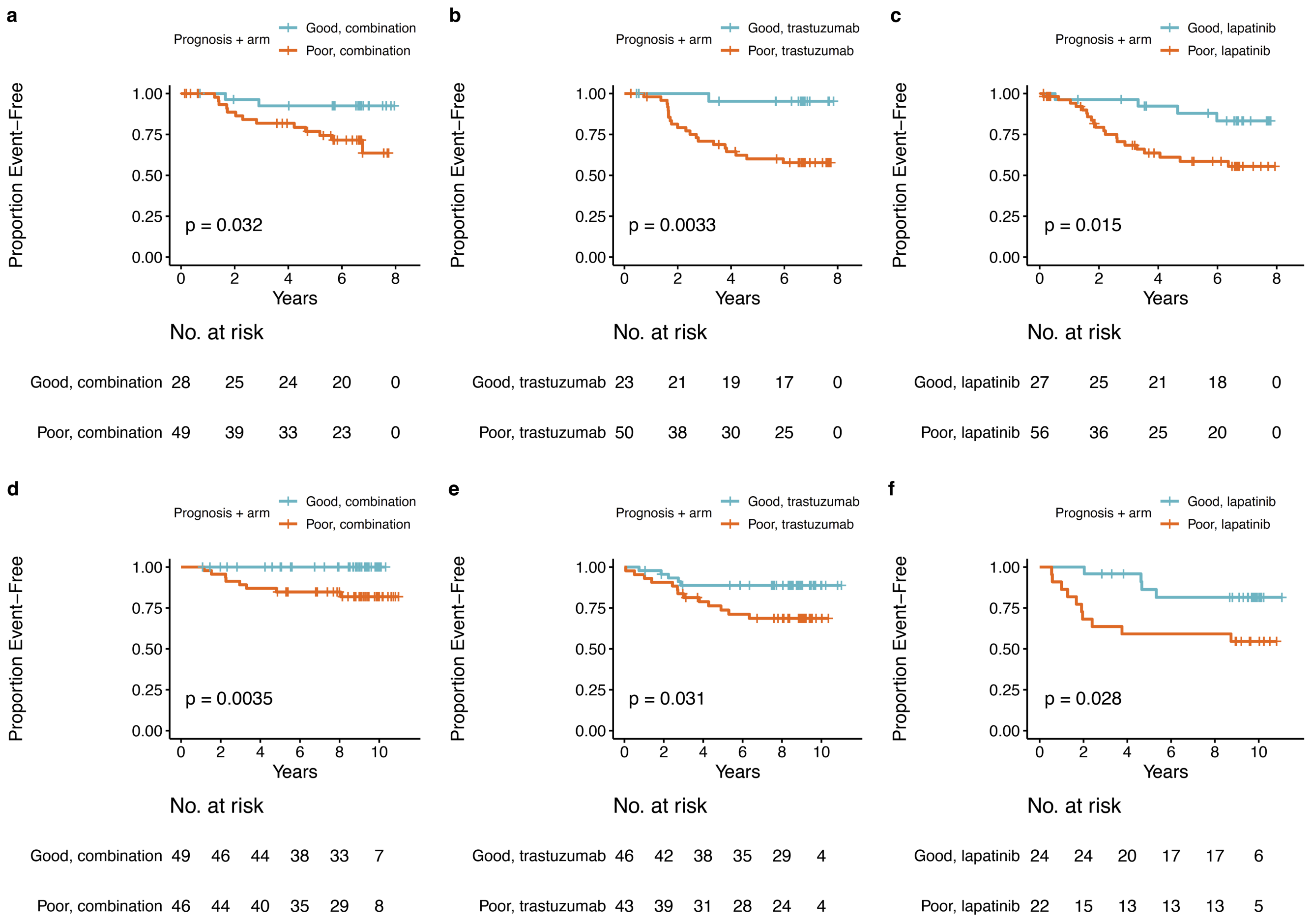

Event-free survival outcomes based on the groups identified by the prognostic model according to treatment arm in the NeoALTTO and CALGB 40601 studies.

a Kaplan–Meier plot showing EFS in the NeoALTTO subgroup with all variables in the model available (breast pCR, hormone receptor status, clinical nodal status, TILs, BCR evenness) and randomized to the anti-HER2 combination arm (added to paclitaxel), i.e., trastuzumab + lapatinib (N = 77). **b** Kaplan–Meier plot showing EFS in the NeoALTTO subgroup with all variables in the model available and randomized to receiving trastuzumab (N = 73). **c** Kaplan–Meier plot showing EFS in the NeoALTTO subgroup with all variables in the model available and randomized to receiving lapatinib (N = 83).

d Kaplan–Meier plot showing EFS in the CALGB 40601 subgroup with all variables in the model available and randomized to the anti-HER2 combination arm, i.e., trastuzumab + lapatinib (N = 95). **e** Kaplan–Meier plot showing EFS in the CALGB 40601 subgroup with all variables in the model available and randomized to receiving trastuzumab (N = 89). **f** Kaplan–Meier plot showing EFS in the CALGB 40601 subgroup with all variables in the model available and randomized to receiving lapatinib (N = 46).

Patients are stratified according to low risk (good prognosis group) and high risk (poor prognosis), based on the score derived from the prognostic model. Patients with a score ≤ -1.3763 were assigned to the good prognosis group, based on the cutoff identified in the NeoALTTO cohort. P values are from log-rank test.

BCR: B cell receptor; EFS: event-free survival; TILs: tumor-infiltrating lymphocytes.

Supplementary Figure 20

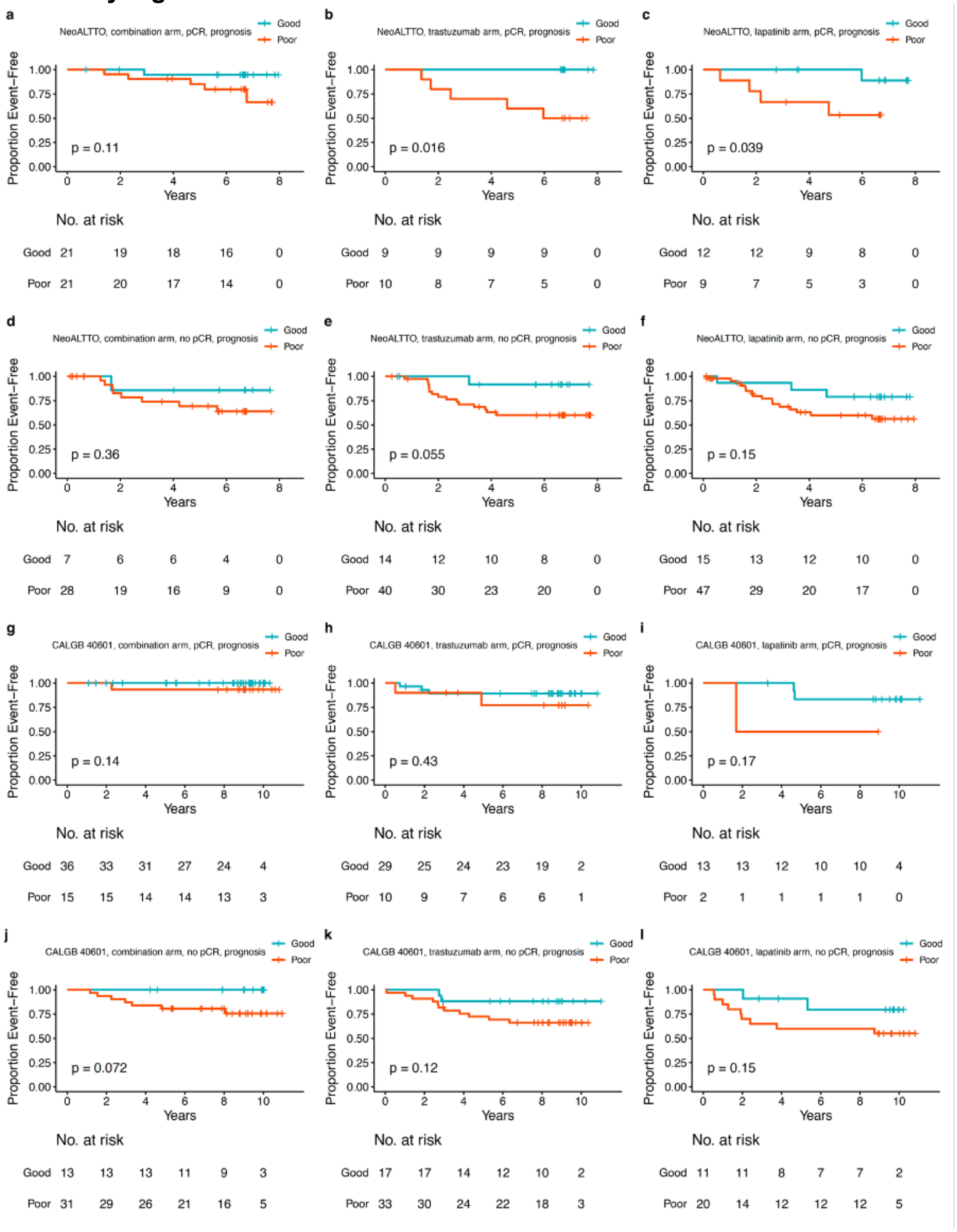

Event-free survival outcomes based on the groups identified by the prognostic model according to pCR status (ypT0/is) and treatment arm in the NeoALTTO and CALGB 40601 studies.

a Kaplan–Meier plot showing EFS in NeoALTTO patients randomized to the anti-HER2 combination arm, i.e., trastuzumab + lapatinib, and achieving pCR (ypT0/is) (N = 42), according to the prognostic groups identified with HER2-EveNT. **b** Kaplan–Meier plot showing EFS in NeoALTTO patients randomized to the trastuzumab arm and achieving pCR (ypT0/is) (N = 19), according to the prognostic groups identified with HER2-EveNT. **c** Kaplan–Meier plot showing EFS in NeoALTTO patients randomized to the lapatinib arm and achieving pCR (ypT0/is) (N = 21), according to the prognostic groups identified with HER2-EveNT.

d Kaplan–Meier plot showing EFS in NeoALTTO patients randomized to the anti-HER2 combination arm, i.e., trastuzumab + lapatinib, and not achieving pCR (N = 35), according to the prognostic groups identified with HER2-EveNT. **e** Kaplan–Meier plot showing EFS in NeoALTTO patients randomized to the trastuzumab arm and not achieving pCR (N = 44), according to the prognostic groups identified with HER2-EveNT. **f** Kaplan–Meier plot showing EFS in NeoALTTO patients randomized to the lapatinib arm and not achieving pCR (N = 62), according to the prognostic groups identified with HER2-EveNT.

g Kaplan–Meier plot showing EFS in CALGB 40601 patients randomized to the anti-HER2 combination arm, i.e., trastuzumab + lapatinib, and achieving pCR (ypT0/is) (N = 51), according to the prognostic groups identified with HER2-EveNT. **h** Kaplan–Meier plot showing EFS in CALGB 40601 patients randomized to the trastuzumab arm and achieving pCR (ypT0/is) (N = 39), according to the prognostic groups identified with HER2-EveNT. **i** Kaplan–Meier plot showing EFS in CALGB 40601 patients randomized to lapatinib arm and achieving pCR (ypT0/is) (N = 15), according to the prognostic groups identified with HER2-EveNT.

j Kaplan–Meier plot showing EFS in CALGB 40601 patients randomized to the anti-HER2 combination arm, i.e., trastuzumab + lapatinib, and not achieving pCR (N = 44), according to the prognostic groups identified with HER2-EveNT. **k** Kaplan–Meier plot showing EFS in CALGB 40601 patients randomized to the trastuzumab arm and not achieving pCR (N = 50), according to the prognostic groups identified with HER2-EveNT. **l** Kaplan–Meier plot showing EFS in CALGB 40601 patients randomized to the lapatinib arm and not achieving pCR (N = 31), according to the prognostic groups identified with HER2-EveNT.

P values are from log-rank test.

EFS: event-free survival; pCR: pathological complete response.

Supplementary Figure 21

a

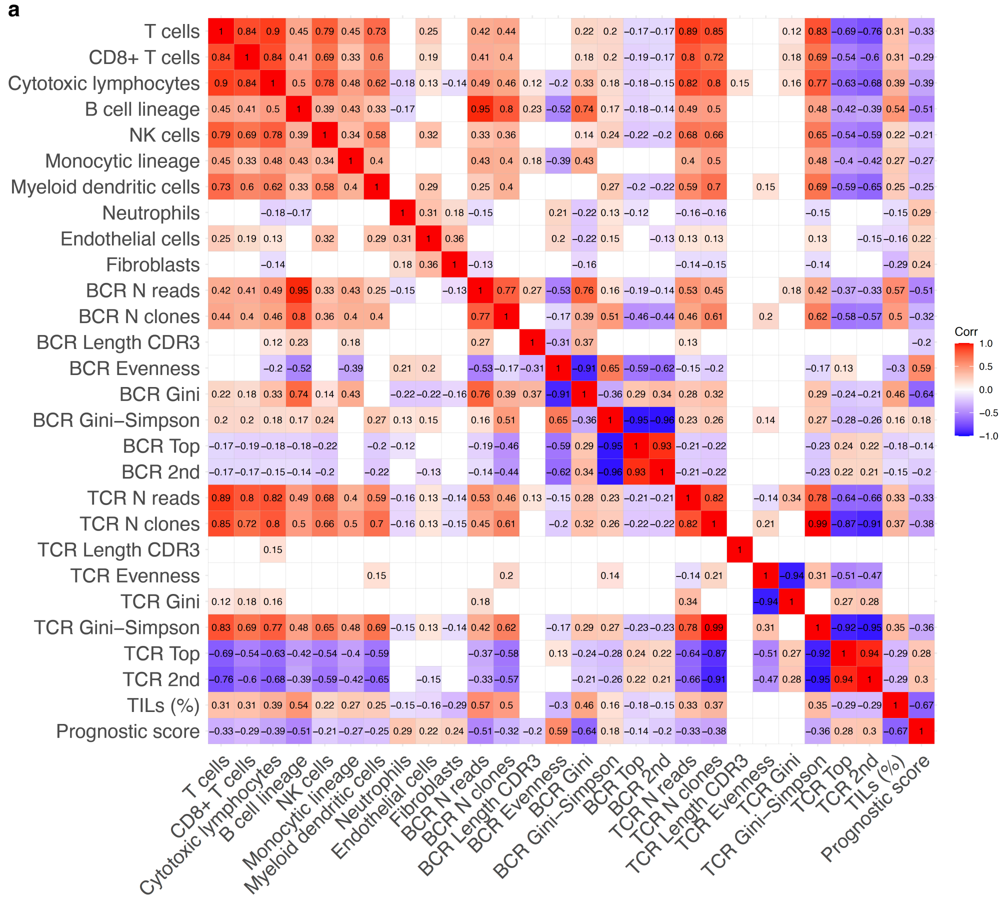

b

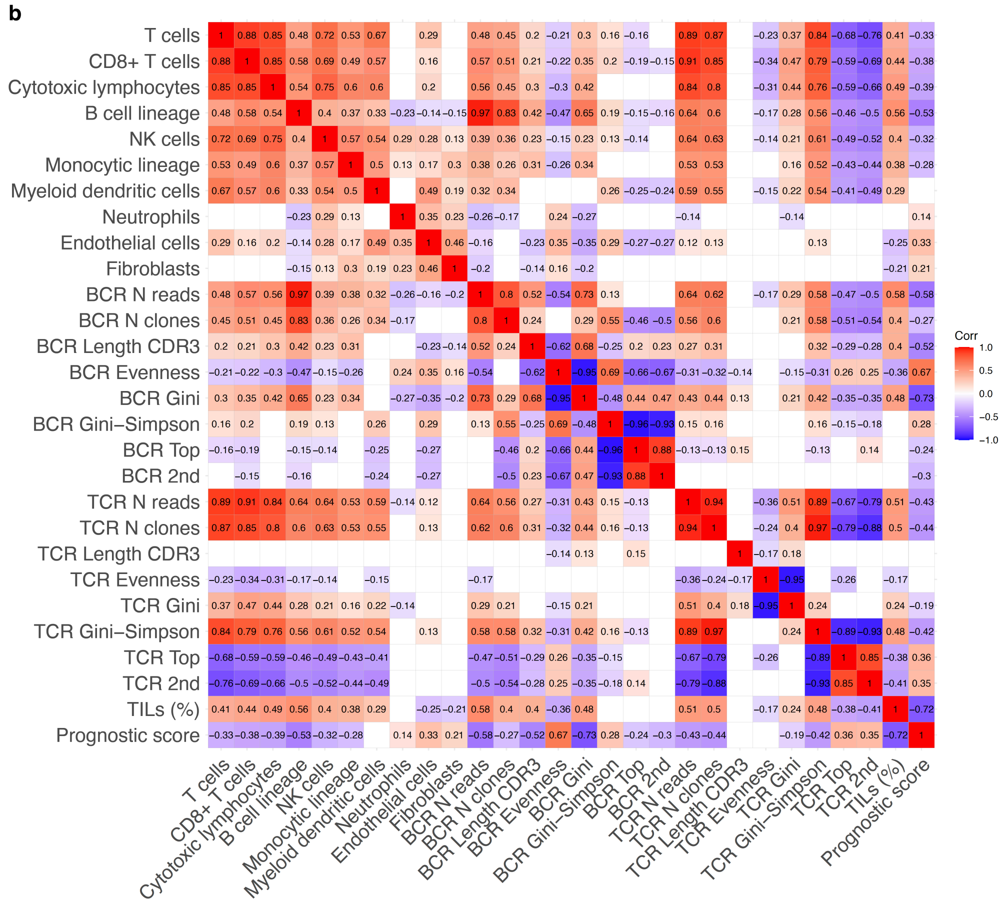

Correlations between immune cells and stromal cells deconvoluted with MCP-counter, BCR/TCR repertoire measures, TIL levels and prognostic score, in NeoALTTO and CALGB 40601.

a Correlations between eight immune populations [T cells (CD3+), CD8+ T cells, cytotoxic lymphocytes, NK cells, B cell lineage, cells originating from monocytes (monocytic lineage), myeloid dendritic cells and neutrophils], and two stromal cell types (endothelial cells and fibroblasts) estimated with MCP-counter, BCR/TCR measures, TILs and prognostic score (HER2-EveNT) in NeoALTTO.

b Correlations between the eight immune populations, endothelial cells and fibroblasts estimated with MCP-counter, BCR/TCR measures, TILs and prognostic score (HER2-EveNT) in CALGB 40601.

Correlations are Spearman (pairwise complete observations). Only correlations with $P < 0.05$ are shown. Correlations and P values available in Supplementary data 17 (a) and 18 (b). The number of reads (N reads) is normalized by the total number of reads mapping to the transcriptome in each sample, and multiplied by 1000.

BCR: B cell receptor; CDR3: complementarity-determining region 3; NK: natural killer; TCR: T cell receptor; TILs: tumor-infiltrating lymphocytes.

Supplementary Figure 22

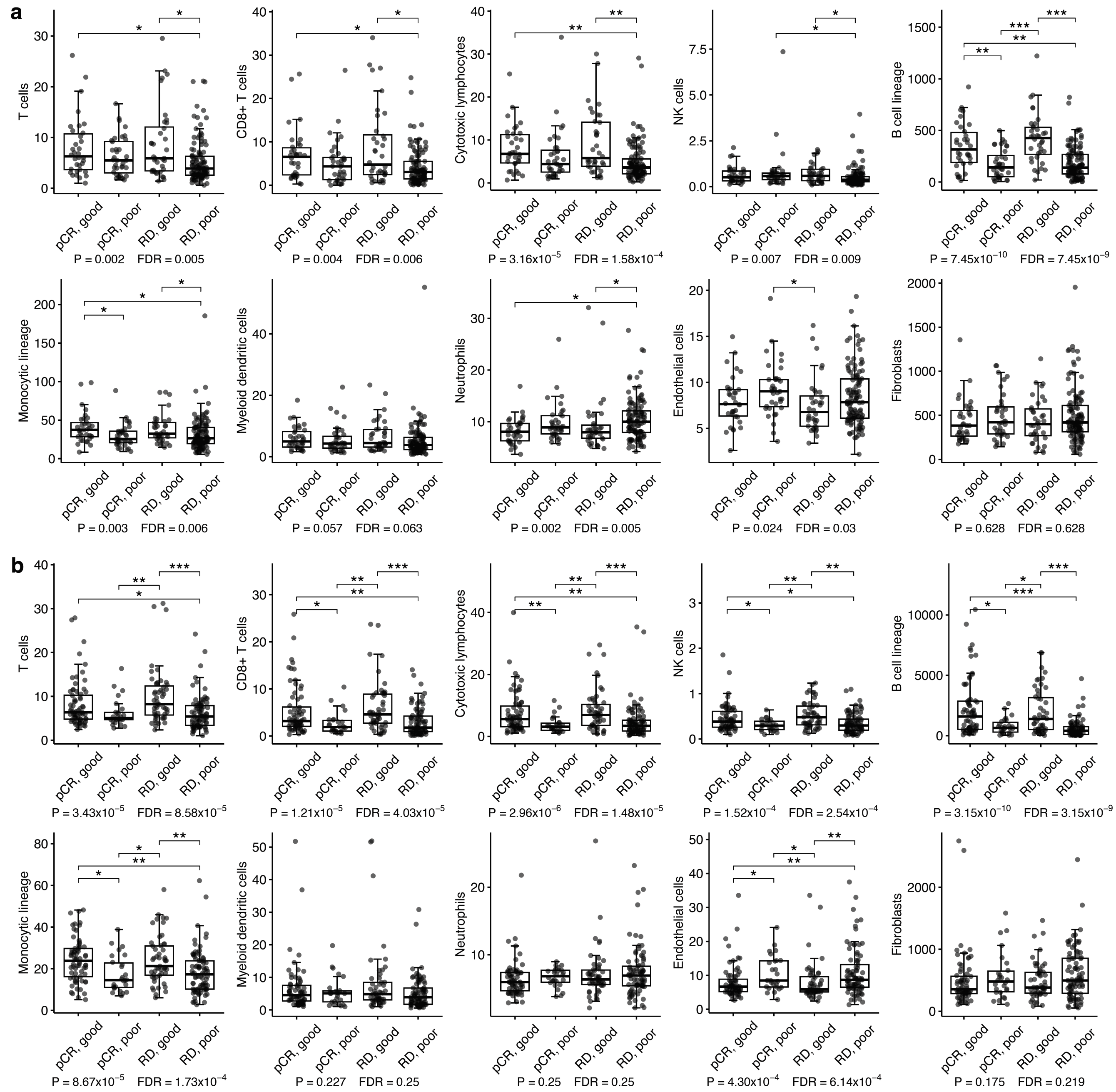

Estimations of immune cell infiltration and stromal cells deconvoluted with MCP-counter, according to pCR (ypT0/is ypN0) status and prognostic groups.

a Estimation of the absolute abundance of eight immune populations [T cells (CD3+), CD8+ T cells, cytotoxic lymphocytes, NK cells, B cell lineage, cells originating from monocytes (monocytic lineage), myeloid dendritic cells and neutrophils], and two stromal cell types (endothelial cells and fibroblasts) according to pCR/RD and prognostic group (good/poor) in NeoALTTO (N = 224).

b Estimation of the absolute abundance of the eight immune populations, endothelial cells and fibroblasts according to pCR/RD and prognostic group (good/poor) in CALGB 40601 (N = 230).

Statistical differences across groups were assessed using Kruskal-Wallis test (P values at the bottom of the panels) and Wilcoxon rank sum test (when comparing one group against each one of the others). FDRs were then obtained adjusting P values using Benjamini & Hochberg method (applied on the comparisons performed in each study, separately). For Wilcoxon tests, FDRs < 0.05 are shown. In the panels: * = FDR < 0.05 and ≥ 0.01; ** = FDR < 0.01 and ≥ 0.001; *** =

FDR < 0.001. P values are two-sided. P values and FDRs available in Supplementary data 19 and 20.

In boxplots, the boxes are defined by the upper and lower quartile; the median is shown as a bold colored horizontal line; whiskers extend to the most extreme data point which is no more than 1.5 times the interquartile range from the box.

BCR: B cell receptor; FDR: false discovery rate; NK: natural killer; pCR: pathological complete response; RD: residual disease.

Supplementary Figure 23

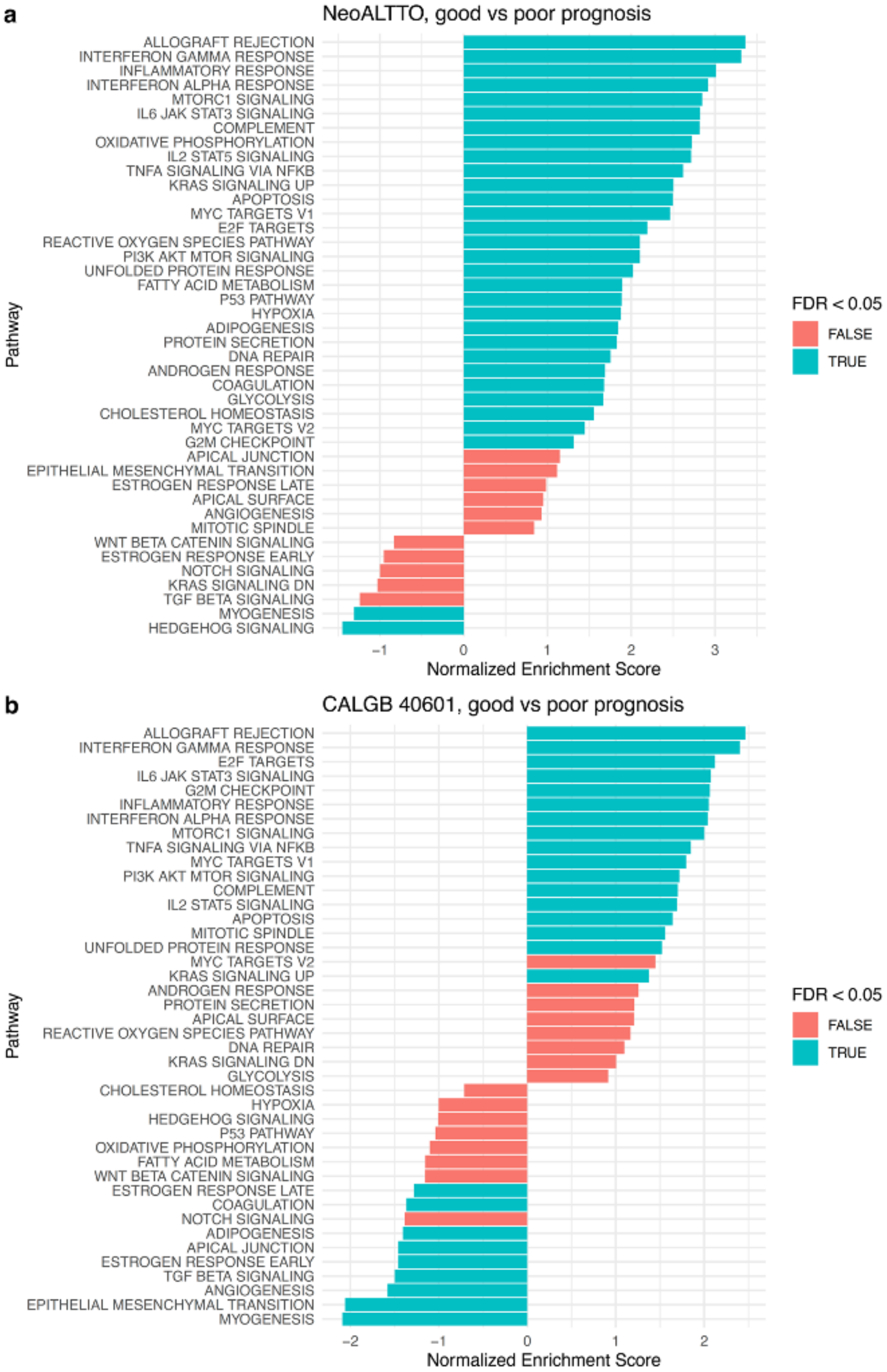

Gene-set enrichment analysis comparing samples classified as good or poor prognosis according to the prognostic score.

a Results from fgsea analysis including 42 hallmark gene sets, showing normalized enrichment scores in NeoALTTO.

b Results from fgsea analysis, showing normalized enrichment scores in CALGB 40601.

Positive scores are associated to an enrichment in the good prognosis group, while negative scores are associated with an enrichment in the poor prognosis group. Pathways are considered significantly enriched for FDR < 0.05. P values are two-sided. Normalized enrichment scores and FDRs available in Supplementary data 26. Source data are available.

FDR = false discovery rate.

Supplementary Figure 24

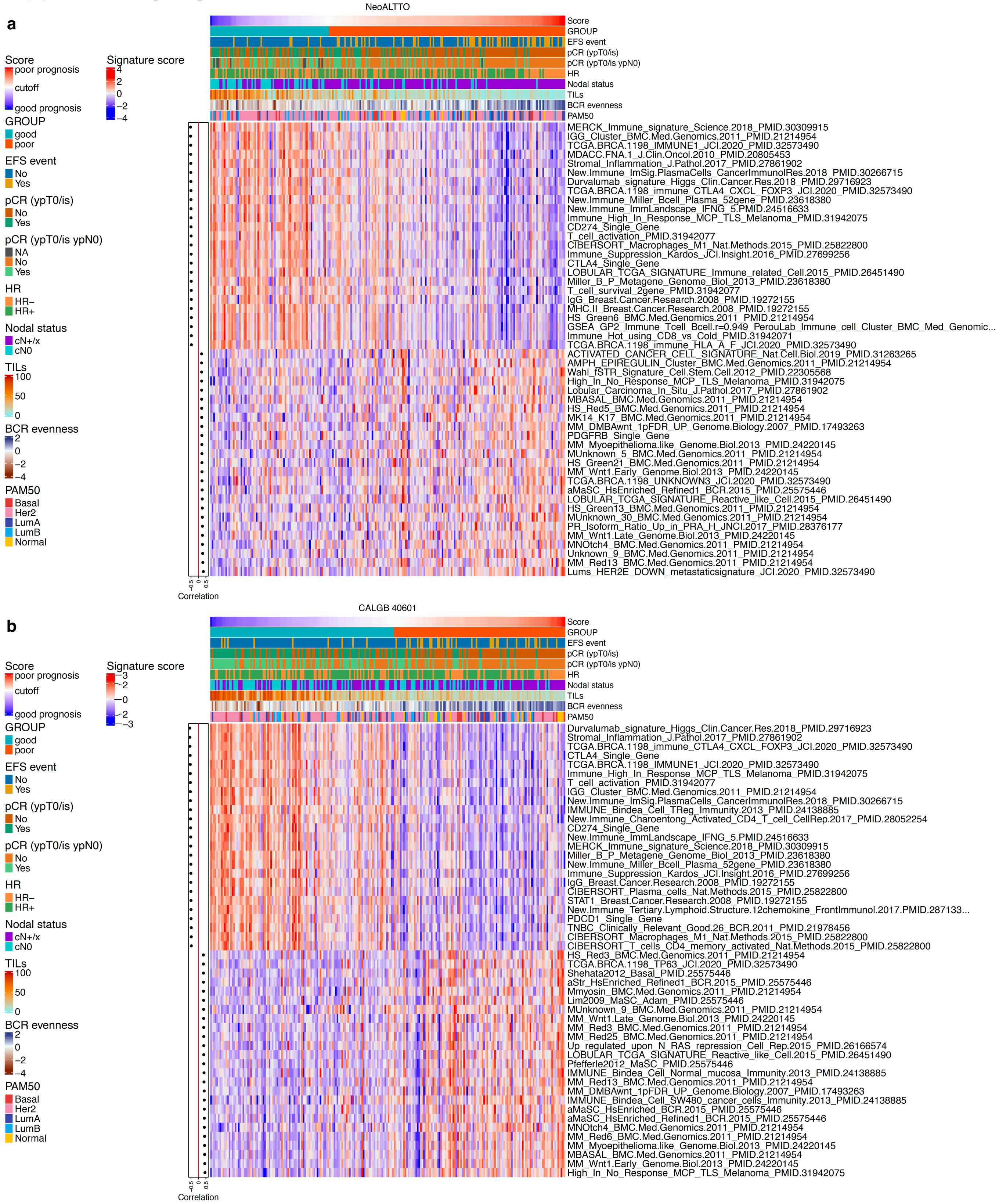

Heatmap of signature scores and correlations with the HER2-EveNT score in the NeoALTTO and CALGB 40601 datasets.

a Heatmap showing the top 25 signatures negatively and positively correlated (total N = 50) with the risk score in the NeoALTTO model cohort (N = 233).

b Heatmap showing the top 25 signatures negatively and positively correlated (total N = 50) with the risk score in the CALGB 40601 model cohort (N = 230).

Annotations on the top part of the heatmap include the HER2-EveNT score as continuous variable (used to order the columns of the heatmap), the prognostic groups, presence or absence of events as defined by EFS, pCR status (according to both breast and breast + axilla definition), hormone receptor status, clinical nodal status, TIL levels (%), BCR evenness (values scaled as used in the model), and PAM50 subtypes.

On the left side, Spearman correlation values between the signature scores and the prognostic score are shown. The red line divides positive and negative correlations. Correlations between gene expression signature scores and HER2-EveNT prognostic score, and P values available in Supplementary data 34 (a) and 35 (b).

Basal: basal-like; BCR: B cell receptor; EFS: event-free survival; Her2: HER2-Enriched; HR: hormone receptor; LumA: luminal A; LumB: luminal B; Normal: normal-like; pCR: pathological complete response.

Supplementary Figure 25

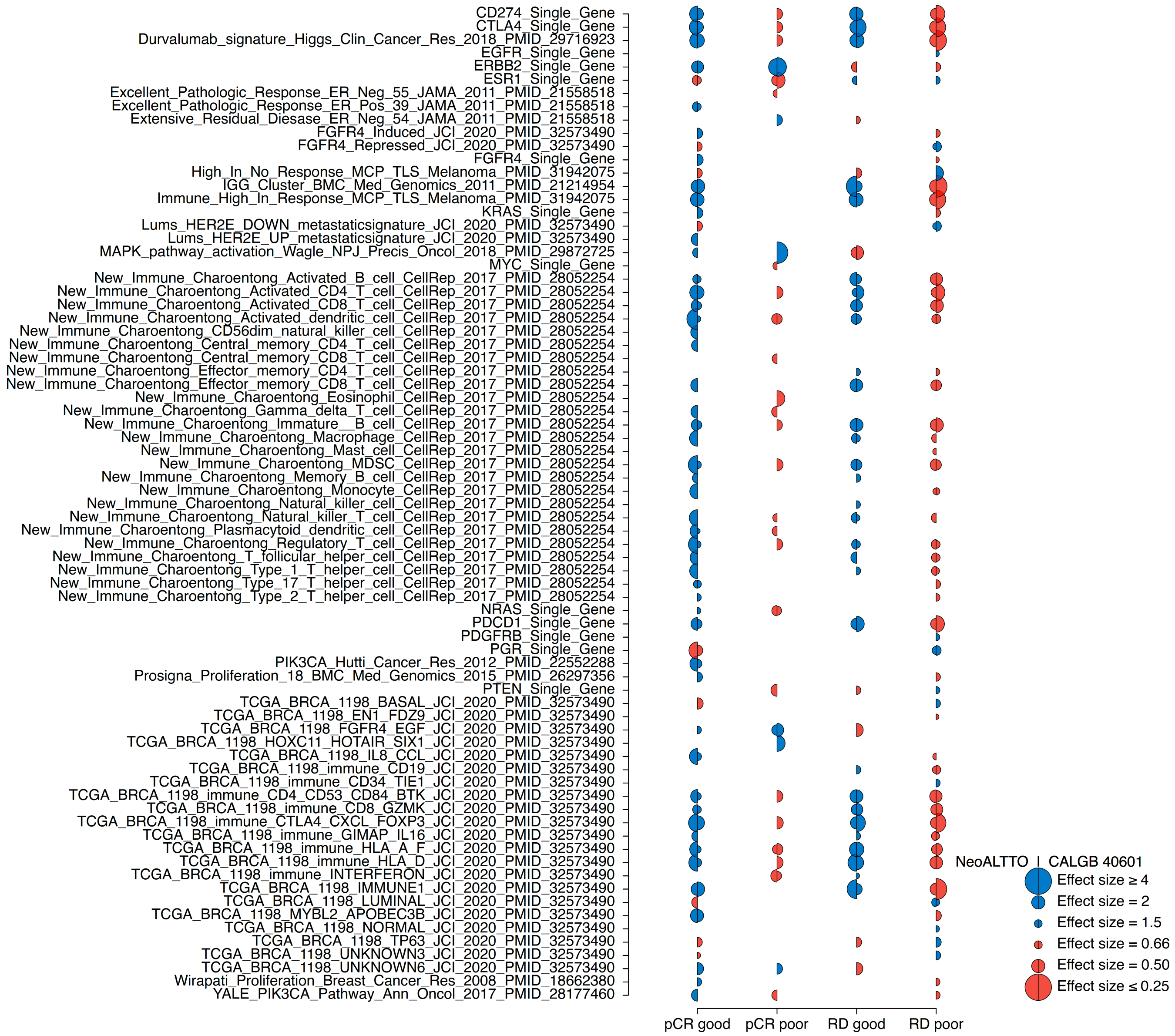

Characterization of the prognostic groups (good vs. poor) according to pCR (ypT0/is ypN0) status using 98 signatures/single genes describing immune related processes, proliferation, and HER2/estrogen/progesterone pathways.

Wilcoxon rank sum test was used to compare each group against the others (i.e., one vs. rest). The effect size represents the direction of the association (positive, blue, if > 1, negative, red, if < 1), and was calculated by applying a linear regression model. FDRs were obtained adjusting P values using Benjamini & Hochberg method (applied on all the comparisons performed for signatures/single genes in each study, separately). P values are two-sided. Only signatures with FDR < 0.05 in at least one group are shown. The dimension of the circle varies proportionally to the effect size (see figure legend). The left and right halves of the circle represent the effect size in NeoALTTO (N = 224) and CALGB 40601 (N = 230), respectively. Effect sizes, P values and FDRs available in Supplementary data 36 (NeoALTTO) and 37 (CALGB 40601).

FDR: false discovery rate; pCR: pathological complete response; RD: residual disease.

SUPPLEMENTARY TABLES 1 and 2

Supplementary Table 1

NeoALTTO	good prognosis (N=78)	poor prognosis (N=155)	p value
**Age**			0.237 (1)
- Median (Q1, Q3)	51 (44.25, 57)	48 (40.5, 56.5)	
**T size**			0.048 (2)
- ≤cT2	54 (69.2%)	86 (55.5%)	
- ≥cT3	24 (30.8%)	69 (44.5%)	
**N stage**			4.24×10^{-10} (2)
- cN+/x	36 (46.2%)	133 (85.8%)	
- cN0	42 (53.8%)	22 (14.2%)	
**HR status**			0.403 (2)
- Negative	32 (41.0%)	74 (47.7%)	
- Positive	46 (59.0%)	81 (52.3%)	
**Breast pCR**			3.96×10^{-5} (2)
- No	36 (46.2%)	115 (74.2%)	
- Yes	42 (53.8%)	40 (25.8%)	
**Breast + axilla pCR**			3.01×10^{-4} (2)
- Not available	8	1	
- No	36 (51.4%)	118 (76.6%)	
- Yes	34 (48.6%)	36 (23.4%)	
**Treatment Arm**			0.814 (2)
- Lapatinib (L)	27 (34.6%)	56 (36.1%)	
- Trastuzumab (T)	23 (29.5%)	50 (32.3%)	
- T + L	28 (35.9%)	49 (31.6%)	
**TILs (%)**			2.50×10^{-17} (1)
- Median (Q1, Q3)	34.375 (20, 50)	7.500 (5, 16.25)	
**BCR evenness (scaled)**			1.32×10^{-13} (1)
- Median (Q1, Q3)	-0.675 (-1.183, -0.225)	0.346 (-0.216, 0.919)	
**PAM50**			0.101 (2)
- HER2-Enriched	53 (67.9%)	89 (57.4%)	
- Basal-like	6 (7.7%)	12 (7.7%)	
- Luminal B	14 (17.9%)	24 (15.5%)	
- Luminal A	4 (5.1%)	17 (11.0%)	
- Normal-like	1 (1.3%)	13 (8.4%)	

Patients' characteristics and pathological complete response rates according to HER2-EveNT prognostic groups in the NeoALTTO cohort (N = 233).

BCR: B cell receptor; HR: hormone receptor; pCR: pathological complete response; Q1: quartile 1; Q3: quartile 3; TILs: tumor-infiltrating lymphocytes. (1) Wilcoxon rank sum test. (2) Fisher's Exact Test. P values are two-sided.

Supplementary Table 2

CALGB 40601	good prognosis (N=119)	poor prognosis (N=111)	p value
**Age**			0.428 (1)
- Median (Q1, Q3)	48 (41, 53)	49 (41, 57)	
**T size**			0.003 (2)
- Not available	5	10	
- ≤cT2	96 (84.2%)	67 (66.3%)	
- ≥cT3	18 (15.8%)	34 (33.7%)	
**N stage**			0.011 (2)
- cN+/x	58 (48.7%)	73 (65.8%)	
- cN0	61 (51.3%)	38 (34.2%)	
**HR status**			0.285 (2)
- Negative	44 (37.0%)	49 (44.1%)	
- Positive	75 (63.0%)	62 (55.9%)	
**Breast pCR**			3.01×10^{-10} (2)
- No	41 (34.5%)	84 (75.7%)	
- Yes	78 (65.5%)	27 (24.3%)	
**Breast + axilla pCR**			2.56×10^{-7} (2)
- No	51 (42.9%)	85 (76.6%)	
- Yes	68 (57.1%)	26 (23.4%)	
**Treatment Arm**			1.000 (2)
- Lapatinib (L)	24 (20.2%)	22 (19.8%)	
- Trastuzumab (T)	46 (38.7%)	43 (38.7%)	
- T + L	49 (41.2%)	46 (41.4%)	
**TILs (%)**			1.19×10^{-20} (1)
- Median (Q1, Q3)	40 (20, 70)	15 (10, 20)	
**BCR evenness (scaled)**			2.95×10^{-21} (1)
- Median (Q1, Q3)	-0.392 (-0.930, 0.088)	0.818 (0.377, 1.366)	
**PAM50**			0.055 (2)
- HER2-Enriched	76 (63.9%)	55 (49.5%)	
- Basal-like	10 (8.4%)	9 (8.1%)	
- Luminal B	17 (14.3%)	15 (13.5%)	
- Luminal A	10 (8.4%)	16 (14.4%)	
- Normal-like	6 (5.0%)	16 (14.4%)	

Patients' characteristics and pathological complete response rates according to HER2-EveNT prognostic groups defined in the CALGB 40601 cohort (N = 230).

BCR: B cell receptor; HR: hormone receptor; pCR: pathological complete response; Q1: quartile 1; Q3: quartile 3; TILs: tumor-infiltrating lymphocytes. (1) Wilcoxon rank sum test. (2) Fisher's Exact Test. P values are two-sided.
