# Supplementary material for: Immunological and clinicopathological features predict HER2-positive breast cancer prognosis in the neoadjuvant NeoALTTO and CALGB 40601 randomized trials
Source: Nat Commun. 2023 Nov 3;14:7053. doi: 10.1038/s41467-023-42635-2 (PMC10624889; doi:10.1038/s41467-023-42635-2)
Supplement: Supplementary file 3 — Reporting Summary [file 41467_2023_42635_MOESM3_ESM.pdf]

nature portfolio

Corresponding author(s): Christos Sotiriou

Last updated by author(s): Sep 21, 2022

Reporting Summary

Nature Portfolio wishes to improve the reproducibility of the work that we publish. This form provides structure for consistency and transparency in reporting. For further information on Nature Portfolio policies, see our Editorial Policies and the Editorial Policy Checklist.

Statistics

For all statistical analyses, confirm that the following items are present in the figure legend, table legend, main text, or Methods section.

n/a	Confirmed	
☐	☒	The exact sample size (*n*) for each experimental group/condition, given as a discrete number and unit of measurement
☐	☒	A statement on whether measurements were taken from distinct samples or whether the same sample was measured repeatedly
☐	☒	The statistical test(s) used AND whether they are one- or two-sided<br>*Only common tests should be described solely by name; describe more complex techniques in the Methods section.*
☐	☒	A description of all covariates tested
☐	☒	A description of any assumptions or corrections, such as tests of normality and adjustment for multiple comparisons
☐	☒	A full description of the statistical parameters including central tendency (e.g. means) or other basic estimates (e.g. regression coefficient) AND variation (e.g. standard deviation) or associated estimates of uncertainty (e.g. confidence intervals)
☐	☒	For null hypothesis testing, the test statistic (e.g. *F*, *t*, *r*) with confidence intervals, effect sizes, degrees of freedom and *P* value noted<br>*Give P values as exact values whenever suitable.*
☒	☐	For Bayesian analysis, information on the choice of priors and Markov chain Monte Carlo settings
☒	☐	For hierarchical and complex designs, identification of the appropriate level for tests and full reporting of outcomes
☐	☒	Estimates of effect sizes (e.g. Cohen's *d*, Pearson's *r*), indicating how they were calculated

Our web collection on statistics for biologists contains articles on many of the points above.

Software and code

Policy information about availability of computer code

Data collection	In NeoALTTO, the clinical data collection tool was the Novartis/Instut Jules Bordet (IJB)/BrEAST-defined case report forms (CRFs). In all cases, subject name was not be collected nor transmitted to Novartis. Subject data necessary for analysis and reporting were entered/transmitted into a validated database or data system. Clinical data management was performed by IJB/BrEAST in accordance with applicable Novartis/BIG/IJB/BrEAST standards and data cleaning procedures. Original CRFs were retained by Novartis/IJB/BrEAST, while the investigator retained a copy. These data were then shared by IJB with Frontier Science, and Frontier Science used SAS to create the analysis datasets.<br>In NeoALTTO, RNA sequencing was performed in the BRIGHTcore Sequencing Facility of the Université Libre de Bruxelles. RNA sequencing data were collected as Fastq files.<br>In CALGB 40601, clinical data were collected and stored by the CALGB (Alliance) Statistics and Data Center, and quality was ensured through data review by the Data Center, the study chairperson, and the surgical co-chairperson. Data collection was conducted by the CALGB (Alliance) Statistics and Data Center by Alliance statisticians using SAS software (version 9.2; SAS Institute, Cary, NC) and R software (version 3.0.1; https://www.r-project.org).<br>In CALGB 40601, RNA sequencing was performed in the University of North Carolina High-Throughput Sequencing Facility. RNA sequencing data were collected as Fastq files.
Data analysis	All statistical analyses in the present retrospective correlative analysis were performed using the R software (v4.0.5). B and T cell receptor (BCR, TCR) repertoires were extracted from RNA sequencing data using the MiXCR tool (v3.0.13). The script to compute BCR/TCR measures is deposited at https://github.com/BCTL-Bordet/BCR_TCR_analyses, and was made available to Reviewers during manuscript submission.<br>The following R packages were used: MCPcounter (v1.2.0); immunedeconv (v2.0.3); tximport (v1.16.0); reldist (v1.6-6); stats (v4.0.5); DESeq2 (v1.28.1); msigdbr (v7.4.1); fgsea (v1.14.0); topGO (v2.40.0); ComplexHeatmap (v2.4.3); GSVA (v1.36.3). For BCR/TCR analyses, immune-deconvolution, differential gene expression, and gene set enrichment analyses (i.e., all analysis specifically conducted in each study separately) starting from BAM files obtained in the NeoALTTO and CALGB 40601 studies, read pairs were trimmed using Trimmomatic v0.39,

and Salmon v1.5.1 was used for alignment to the human reference GRCh38/hg38 (patch 13), using GENCODE v38 for the gene positions. Intrinsic subtypes were obtained from a study-effect corrected analysis of the RNA sequencing data. Reads were aligned to the human reference GRCh38/hg38 genome using STAR v2.4.2a. Transcript (GENCODE v22) abundance estimates were generated by Salmon v0.6.0 in '-quant' mode, based on the STAR alignments.

For manuscripts utilizing custom algorithms or software that are central to the research but not yet described in published literature, software must be made available to editors and reviewers. We strongly encourage code deposition in a community repository (e.g. GitHub). See the Nature Portfolio guidelines for submitting code & software for further information.

Data

Policy information about availability of data

All manuscripts must include a data availability statement. This statement should provide the following information, where applicable:

- Accession codes, unique identifiers, or web links for publicly available datasets
- A description of any restrictions on data availability
- For clinical datasets or third party data, please ensure that the statement adheres to our policy

For reproducibility purposes, the RNA sequencing data (fastq files) at baseline from NeoALTTO have been deposited to the European Genome-phenome Archive (EGA) database (https://ega-archive.org) and assigned the accession code EGAS00001007563 (https://ega-archive.org/studies/EGAS00001007563). The data can be obtained upon signature of a data access agreement between the investigator requesting the access and Institut Jules Bordet (IJB), subject to applicable laws. For reproducibility purposes, the NeoALTTO clinical data at IJB can be obtained upon signature of a data transfer agreement between the investigator and IJB, subject to applicable laws. The access to these data can be requested by contacting the corresponding Author (christos.sotiriou@hubruxelles.be).

For investigators aiming to perform original research, the NeoALTTO RNA sequencing data at baseline and the clinical data are available upon request after submission of a research project proposal (RPP) to the RPP's administrator (alttoresearchproposals@frontier-science.co.uk). In detail, access to data for research will be granted upon review of the RPP and its endorsement by the study Steering Committee, and after entering into an appropriate data access agreement between BIG, IJB, and the investigator, subject to applicable laws. More details and documents required can be found at https://bigagainstbreastcancer.org/clinical-trials/neoaltto/ under the section "Translational Research".

The policy for access to residual biological samples and data in the NeoALTTO study is a fair scientific review process set up to ensure precious biological samples or data collected in the studies are accessed appropriately, to avoid duplication of efforts and foster collaboration. The data from the study are not anonymized yet, only pseudonymized, therefore they are still considered identifiable, and cannot be made publicly available at this point. In order to ensure that they are shared in a way that preserves the privacy of patients and complies with the relevant laws and regulations including the European General Data Protection Regulation (GDPR), researchers can only access the data after they sign the data transfer agreements mentioned above, either for reproducibility or for original research purposes.

Gene expression data from CALGB 40601 is deposited in Gene Expression Omnibus (GEO) (https://www.ncbi.nlm.nih.gov/geo/) (accession code GSE116335) (https://www.ncbi.nlm.nih.gov/geo/query/acc.cgi?acc=GSE116335). Fastq files (RNA sequencing) and clinical data, including TIL levels, from CALGB 40601 are available at the NCBI database of Genotypes and Phenotypes (dbGaP) repository (https://www.ncbi.nlm.nih.gov/gap/) (accession code phs001570.v3.p1) (https://www.ncbi.nlm.nih.gov/projects/gap/cgi-bin/study.cgi?study_id=phs001570.v3.p1). The data are available under controlled access to ensure that they are shared in a manner consistent with the research participants' informed consent, and that the confidentiality of the data and the privacy of participants is protected. Principal investigators seeking access to dbGaP datasets have to request them through the controlled-access portal. More information can be found at https://dbgap.ncbi.nlm.nih.gov/aa/wga.cgi?page=login.

Clinicopathological data from NeoALTTO and CALGB 40601 can be obtained as specified above. Other data supporting the findings of this study are available within the article, supplementary information files, supplementary data and source data provided with this paper. MSigDB is available at https://www.gsea-msigdb.org/gsea/msigdb.

Human research participants

Policy information about studies involving human research participants and Sex and Gender in Research.

Reporting on sex and gender	The NeoALTTO and CALGB 40601 trials recruited women affected by HER2-positive breast cancer.
Population characteristics	Patients' characteristics of this translational research study are reported in the manuscript, Table 1.
Recruitment	This is a retrospective translational research study conducted in the context of the NeoALTTO and CALGB 40601 clinical trials. The recruitment of patients in the two clinical trials has been previously described in the original NeoALTTO (PMID: 22257673) and CALGB 40601 (PMID: 26527775) publications.<br>The clinical investigators at site selected the patients for screening. The study teams are unaware whether there was any self selection bias in that area. Even if there was a bias in terms of the people who volunteered for the trial, the fact that they were randomized to treatment group should mean they were balanced across the arms in that sense.<br>For NeoALTTO, the full list of eligibility criteria is available at https://clinicaltrials.gov/study/NCT00553358. Key eligibility criteria included age ≥18 years, histologically confirmed HER2-positive early breast cancer, tumor size greater than 2 cm, adequate hepatic, renal, cardiac, and bone marrow function at baseline, and left ventricular ejection fraction at baseline of 50% or more.<br>For CALGB 40601, the full list of eligibility criteria is available at https://clinicaltrials.gov/study/NCT00770809. Patients eligible for CALGB 40601 had newly diagnosed, histologically confirmed, untreated clinical stage II to III HER2-positive disease. Patients were age ≥ 18 years, had tumors ≥ 1 cm in size, and had a pretreatment left ventricular ejection fraction ≥ 50%.<br>The cohorts with RNA sequencing data available have been previously described for NeoALTTO (PMID: 27684533) and CALGB 40601 (PMID: 26527775, PMID: 33095682).
Ethics oversight	Ethics committee and relevant health authorities at each participating site approved the NeoALTTO and CALGB 40601 studies and all patients provided written informed consent including future biomarker research. For NeoALTTO, the the sites which run the trial are listed in Supplementary data 38. For the CALGB 40601 trial, the protocol was central IRB approved, being the University of North Carolina the lead Institution and Dr. Lisa Carey the study chair.

The current combined analysis has been approved by the TransALTTO scientific committee and Alliance Publications Committee, and was conducted in accordance to the Declaration of Helsinki.

Note that full information on the approval of the study protocol must also be provided in the manuscript.

Field-specific reporting

Please select the one below that is the best fit for your research. If you are not sure, read the appropriate sections before making your selection.

- [x] Life sciences
- [ ] Behavioural & social sciences
- [ ] Ecological, evolutionary & environmental sciences

For a reference copy of the document with all sections, see nature.com/documents/nr-reporting-summary-flat.pdf

Life sciences study design

All studies must disclose on these points even when the disclosure is negative.

Sample size

Patients included in the present manuscript represent a subgroup with RNA sequencing data available, as previously described for NeoALTTO, N=254 (PMID: 27684533) and CALGB 40601, N=264 (PMID: 33095682).

Data exclusions

No data were excluded.

Replication

Findings were assessed in NeoALTTO and independently validated in CALGB 40601. In detail, BCR and TCR repertoires were computed independently in the two trials and associations with hormone receptor status, PAM50 subtypes and clinical outcomes were analyzed separately. The prognostic score derived in NeoALTTO was independently validated in CALGB 40601.

Randomization

Patients included in the retrospective correlative translational research analysis in the current manuscript are a subset with RNA sequencing data available, as previously described for NeoALTTO (PMID: 22257673) and CALGB 40601 (PMID: 26527775), and received randomized anti-HER2 treatments (trastuzumab, lapatinib, trastuzumab + lapatinib) as described in the original publications for the two trials (NeoALTTO PMID: 22257673; CALGB 40601 PMID: 26527775). The distribution of the treatments in the NeoALTTO and CALGB 40601 RNA sequencing cohorts included in the manuscript is reported in Table 1.

In the NeoALTTO trial, Sites used Frontier Science's web-based randomization system to register patients and receive treatment assignments (Frontier Sciences (www.fstrf.org/Portal) for enrolment version: Subject enrolment system (SES) 2.1.6 to 2.4.1 / RAND 3.34 to 3.37). Treatment allocation was by stratified, permuted blocks randomisation. Block size was six (three groups randomly assigned in a 2:2:2 ratio) and was masked from any individuals actively participating in the trial. Four stratification factors were used: hormone-receptor status (oestrogen-receptor or progesterone-receptor positive, or both, vs both oestrogen-receptor and progesterone-receptor negative); clinical involvement of axillary lymph nodes (N0–1 vs ≥N2); clinical tumour size (T2 [2–5 cm diameter]) vs ≥T3 [>5 cm diameter]); and suitability for breast-conserving surgery (yes vs no). Randomisation was done centrally at the Frontier Science and Technology Research Foundation Randomisation Center, which was accessed by participating sites using a web-based system so that patients were enrolled before the treatment assignment was revealed.

In the CALGB 40601 trial, Sites used the CALGB Web-based Patient Registration system. Randomization was accepted only through CALGB Main Member Institutions, selected affiliate institutions and CCOPs using the Web-based Patient Registration system. Patients were initially randomly assigned with equal probability to the three study arms, and randomized according to the stratification factors: Pretreatment clinical stage: 1) stage II, 2) stage III; Hormone receptor status as defined by local laboratory standards: 1) ER and/or PgR positive, 2) ER and PgR negative. In December 2010, two neoadjuvant studies were presented at the Cancer Therapy and Research Center-American Association for Cancer Research (CTRC-AACR) San Antonio Breast Cancer Symposium that affected the scientific and practical enthusiasm for the TL arm: Geparquinto and NeoALTTO trial. Based on the inferior results and higher toxicity of the investigational lapatinib arms of these studies and in discussion with CTEP, in January 2011 it was decided to amend CALGB 40601 to omit Arm 3 (TL).

Blinding

This is a retrospective correlative analysis in the context of already published clinical trials. As such, no blinding was performed for the analyses in the present manuscript.

Reporting for specific materials, systems and methods

We require information from authors about some types of materials, experimental systems and methods used in many studies. Here, indicate whether each material, system or method listed is relevant to your study. If you are not sure if a list item applies to your research, read the appropriate section before selecting a response.

Materials & experimental systems

n/a	Involved in the study	
x		Antibodies
x		Eukaryotic cell lines
x		Palaeontology and archaeology
x		Animals and other organisms
	x	Clinical data
x		Dual use research of concern

Methods

n/a	Involved in the study	
x		ChIP-seq
x		Flow cytometry
x		MRI-based neuroimaging

Clinical data

Policy information about clinical studies

All manuscripts should comply with the ICMJE guidelines for publication of clinical research and a completed CONSORT checklist must be included with all submissions.

Clinical trial registration	The NeoALTTO trial is registered at www.clinicaltrials.gov as NCT00553358, while the CALGB 40601 trial is registered at www.clinicaltrials.gov as NCT00770809.
Study protocol	The NeoALTTO (NCT00553358) study protocol has been previously described in details at PMID: 22257673, PMID: 25130998 and PMID: 31377477. The CALGB 40601 (NCT00770809) study protocol has been previously described in details at PMID: 26527775 and PMID: 33095682.
Data collection	Clinical data were collected as previously reported in the original trial publications for NeoALTTO (PMID: 22257673) and CALGB 40601 (PMID: 26527775). Statistical analysis was based on the data lock on 26 May 2016 in the NeoALTTO trial and 06 October 2021 in the CALGB 40601 trial. Median follow-up for the NeoALTTO RNA sequencing cohort was 6.7 years, while for CALGB 40601 it was 9.1 years.<br>In NeoALTTO, clinical data were collected at Institut Jules Bordet (Bruxelles, Belgium) and Frontier Science (Scotland, UK).<br>In CALGB 40601, clinical data were collected at the CALGB (Alliance) Statistics and Data Center, Durham, NC.<br>RNA sequencing data were generated and collected as described in PMID: 27684533 for NeoALTTO, and in PMID: 33095682 and PMID: 26527775 for CALGB 40601.<br>In NeoALTTO, translational baseline tumor biopsy (minimum 1 core formalin–fixed paraffin–embedded, 2 cores snap frozen) could be up to 4 weeks prior to randomization. RNA sequencing was performed in the BRIGHTcore Sequencing Facility of the Université Libre de Bruxelles.<br>In CALGB 40601, for RNA sequencing analysis pre-treatment tumor samples were collected from all patients prior to initiating protocol chemotherapy using the specimen collection/shipping kits provided by Alliance. The required tumor biopsies include two core biopsies in RNA Later and two core biopsies in 10% neutral buffered formalin to be used for the correlative analyses including RNA sequencing. RNA sequencing was performed in the University of North Carolina High-Throughput Sequencing Facility.<br>The analyses in the present manuscript were performed at the Institut Jules Bordet/Université Libre de Bruxelles and Lineberger Comprehensive Cancer Center/University of North Carolina.
Outcomes	In this retrospective correlative analysis, we evaluated the outcomes pathological complete response (pCR), event-free survival (EFS) and overall survival (OS), which were available in the NeoALTTO trial.<br>Pathological complete response was defined as either absence of invasive tumor cells in the breast (ypT0/is) at surgery, as defined by the National Surgical Adjuvant Breast and Bowel Project (NSABP) criteria, or the absence of invasive tumor cells in the breast and in the axillary lymph nodes (ypT0/is ypN0) according to the Food and Drug Administration (FDA) criteria. These definitions of pCR were applied in both NeoALTTO and CALGB 40601 trials. Pathological complete response defined as ypT0/is was the primary endpoint in both NeoALTTO and CALGB 40601.<br>Secondary endpoints in the NeoALTTO trial included EFS and OS. In NeoALTTO, EFS was defined as the time from randomization to first event, i.e., breast cancer relapse after surgery, second primary malignant neoplasm, or death without recurrence for women who received surgery for breast cancer, or, for those who did not undergo surgery, death during clinical follow-up or non-completion of any neoadjuvant investigational drugs due to progressive disease. To make survival outcomes comparable between the two studies, EFS was also assessed in the CALGB 40601 using the same definition adopted in the NeoALTTO study. Overall survival (OS) was defined as the time from randomization to death from any cause in both NeoALTTO and CALGB 40601.
