# Supplementary material for: Immunological and clinicopathological features predict HER2-positive breast cancer prognosis in the neoadjuvant NeoALTTO and CALGB 40601 randomized trials
Source: Nat Commun. 2023 Nov 3;14:7053. doi: 10.1038/s41467-023-42635-2 (PMC10624889; doi:10.1038/s41467-023-42635-2)
Supplement: Supplementary file 4 — Description of Additional Supplementary Files [file 41467_2023_42635_MOESM4_ESM.pdf]

Description of Additional Supplementary Files

Supplementary Data 1 - 14

Description:

- **Supplementary data 1**: Comparisons of BCR/TCR read and clone counts in NeoALTTO (100-bp and 50-bp) and CALGB 40601, and lengths of CDR3 regions (in nucleotides) for each BCR/TCR chain. P values from the Wilcoxon rank sum test are two-sided.
- **Supplementary data 2**: Anonymized BCR/TCR, single chains and immunoglobulin isotypes measures in NeoALTTO. Absolute number of reads (Nreads) and the number of reads normalized by the total reads mapping to the transcriptome in each sample multiplied by a factor of 1000 (Nreads_NORM) are reported, together with the other measures.
- **Supplementary data 3**: Anonymized BCR/TCR, single chains and immunoglobulin isotypes measures in CALGB 40601. Absolute number of reads (Nreads) and the number of reads normalized by the total reads mapping to the transcriptome in each sample multiplied by a factor of 1000 (Nreads_NORM) are reported, together with the other measures.
- **Supplementary data 4**: Correlation values (Spearman) and P values for BCR/TCR, single chains and TIL levels in NeoALTTO (pairwise complete observations). All correlations between BCR/TCR measures are reported. The number of reads (N reads) is normalized by the total number of reads mapping to the transcriptome in each sample, and multiplied by 1000. P values are two-sided and unadjusted for multiple testing.
- **Supplementary data 5**: Correlation values (Spearman) and P values for BCR/TCR, single chains and TIL levels in CALGB 40601 (pairwise complete observations). All correlations between BCR/TCR measures are reported. The number of reads (N reads) is normalized by the total number of reads mapping to the transcriptome in each sample, and multiplied by 1000. P values are two-sided and unadjusted for multiple testing.
- **Supplementary data 6**: Correlation values (Spearman) and P values for BCR and IgG, IgM, and IgA isotypes in NeoALTTO (pairwise complete observations). Correlations related to number of reads, number of clones, evenness and Gini index measured in BCR and immunoglobulin isotypes are reported. The number of reads (N reads) is normalized by the total number of reads mapping to the transcriptome in each sample, and multiplied by 1000. P values are two-sided and unadjusted for multiple testing.
- **Supplementary data 7**: Correlation values (Spearman) and P values for BCR and IgG, IgM, and IgA isotypes in CALGB 40601 (pairwise complete observations). Correlations related to number of reads, number of clones, evenness and Gini index measured in BCR and immunoglobulin isotypes are reported. The number of reads (N reads) is normalized by the total number of reads mapping to the transcriptome in each sample, and multiplied by 1000. P values are two-sided and unadjusted for multiple testing.
- **Supplementary data 8**: Comparisons (Wilcoxon rank sum test) of BCR/TCR measures in NeoALTTO and CALGB 40601 according to hormone receptor

status (hormone receptor-negative [HR-] vs. hormone receptor-positive [HR+]). P values are two-sided, FDRs are also reported.

- **Supplementary data 9**: Anonymized PAM50 subtypes in NeoALTTO and in CALGB 40601.
- **Supplementary data 10**: Comparisons (Kruskal-Wallis test) of BCR/TCR measures in NeoALTTO and in CALGB 40601 across PAM50 subtypes. P values are two-sided, FDRs are also reported.
- **Supplementary data 11**: Comparisons (Wilcoxon rank sum test) of BCR/TCR measures in NeoALTTO and in CALGB 40601 across PAM50 subtypes. P values are two-sided, FDRs are also reported.
- **Supplementary data 12**: Forward stepwise model to predict event-free survival with Akaike Information Criteria (AIC), showing AIC values and variables tested, as well as table with coefficients and hazard ratios for the variables selected in the model.
- **Supplementary data 13**: HER2-EveNT score, tertiles, and prognostic groups in the NeoALTTO cohort (N = 233 with available score).
- **Supplementary data 14**: HER2-EveNT score, groups defined applying cutoffs from NeoALTTO tertiles, and prognostic groups in the CALGB 40601 cohort (N = 230 with available score).

Supplementary Data 15 - 20

Description:

- **Supplementary data 15**: Anonymized MCP-counter results in NeoALTTO.
- **Supplementary data 16**: Anonymized MCP-counter results in CALGB 40601.
- **Supplementary data 17**: Spearman correlations between MCP-counter cell types, BCR/TCR measures, TILs and the prognostic score in NeoALTTO (using pairwise complete observations). P values are two-sided and unadjusted for multiple testing.
- **Supplementary data 18**: Spearman correlations between MCP-counter cell types, BCR/TCR measures, TILs and the prognostic score in CALGB 40601 (using pairwise complete observations). P values are two-sided and unadjusted for multiple testing.
- **Supplementary data 19**: Comparisons (Kruskal-Wallis test) of MCP-counter measures in NeoALTTO (N = 224) and in CALGB 40601 (N = 230) across groups defined by pCR (ypT0/is ypN0) status and prognostic groups. P values are two-sided, FDRs are also reported.
- **Supplementary data 20**: Comparisons (Wilcoxon rank sum test) of MCP-counter measures in NeoALTTO (N = 224) and in CALGB 40601 (N = 230) across groups defined by pCR (ypT0/is ypN0) status and prognostic groups. P values are two-sided, FDRs are also reported.

Supplementary Data 21 - 31

Description:

- **Supplementary data 21**: Anonymized gene set variation analysis (GSVA) scores computed on samples from patients with available HER2-EveNT score in NeoALTTO (N = 233).
- **Supplementary data 22**: Anonymized gene set variation analysis (GSVA) scores computed on samples from patients with available HER2-EveNT score in CALGB 40601 (N = 230).
- **Supplementary data 23**: Spearman correlations between 42 hallmark gene sets scores calculated at the single-sample level following gene set variation analysis, performed in NeoALTTO (N = 233) and in CALGB 40601 (N = 230), and HER2-EveNT prognostic score. P values are two-sided and unadjusted for multiple testing.
- **Supplementary data 24**: Results from differential expression analysis in NeoALTTO (N = 233), comparing patients with good vs. poor prognosis according to our model. P values are two-sided and adjusted P values ("padj", i.e., FDR according to Benjamini & Hochberg method) are also reported.
- **Supplementary data 25**: Results from differential expression analysis in CALGB 40601 (N = 230), comparing patients with good vs. poor prognosis according to our model. P values are two-sided and adjusted P values ("padj", i.e.,FDR according to Benjamini & Hochberg method) are also reported.
- **Supplementary data 26**: Results from gene set enrichment analysis on 42 hallmark gene sets performed in NeoALTTO (N = 233) and in CALGB 40601 (N = 233), comparing patients with good vs. poor prognosis according to our model. P values are two-sided and adjusted P values ("padj", i.e., FDR according to Benjamini & Hochberg method) are also reported. ES: enrichment score; NES: enrichment score normalized to mean enrichment of random samples of the same size; log_2err: expected error for the standard deviation of the P-value logarithm.
- **Supplementary data 27**: Gene ontology analysis showing biological processes in upregulated genes [FDR < 0.05, log_2(fold change) > 0.58] in NeoALTTO (N = 233) in the good prognosis group, compared to the poor prognosis one. Top 30 processes are shown.
- **Supplementary data 28**: Gene ontology analysis showing biological processes in upregulated genes [FDR < 0.05, log_2(fold change) > 0.58] in CALGB 40601 (N = 230) in the good prognosis group, compared to the poor prognosis one. Top 30 processes are shown.
- **Supplementary data 29**: Gene ontology analysis showing biological processes in downregulated genes [FDR < 0.05, log_2(fold change) < -0.58] in NeoALTTO (N = 233) in the good prognosis group, compared to the poor prognosis one. Top 30 processes are shown.
- **Supplementary data 30**: Gene ontology analysis showing biological processes in downregulated genes [FDR < 0.05, log_2(fold change) < -0.58] in CALGB 40601 (N = 230) in the good prognosis group, compared to the poor prognosis one. Top 30 processes are shown.

- **Supplementary data 31**: Genes with FDR < 0.05 and log_2(fold change) of either > 0.58 or < -0.58 (up- or down-regulated in the good prognosis group as compared to the poor prognosis one, respectively) in both NeoALTTO and CALGB 40601.

Supplementary Data 32 - 37

Description:

- **Supplementary data 32**: Anonymized scores for a pool of 709 gene expression signatures in NeoALTTO.
- **Supplementary data 33**: Anonymized scores for a pool of 709 gene expression signatures in CALGB 40601.
- **Supplementary data 34**: Spearman correlations between a pool of 709 gene expression signature scores and single genes expression levels with the HER2-EveNT prognostic score in NeoALTTO (N = 233). P values are two-sided and unadjusted for multiple testing.
- **Supplementary data 35**: Spearman correlations between a pool of 709 gene expression signature scores and single genes expression levels with the HER2-EveNT prognostic score in CALGB 40601 (N = 230). P values are two-sided and unadjusted for multiple testing.
- **Supplementary data 36**: Comparisons between groups defined by pCR (ypT0/is ypN0) status and prognostic groups in NeoALTTO (N = 224), using a set of 98 gene expression signatures. Each group is compared with the remaining three. For each comparison, effect size and 95% confidence interval (derived from a linear regression model), as well as P value (two-sided, derived from a Wilcoxon rank sum test) and FDR are shown.
- **Supplementary data 37**: Comparisons between groups defined by pCR (ypT0/is ypN0) status and prognostic groups in CALGB 40601 (N = 230), using a set of 98 gene expression signatures. One group is compared with the remaining three. For each comparison, effect size and 95% confidence interval (derived from a linear regression model), as well as P value (two-sided, derived from a Wilcoxon rank sum test) and FDR are shown.

Supplementary Data 38 - 40

Description:

- **Supplementary data 38**: List of sites which run the NeoALTTO clinical trial.
- **Supplementary data 39**: Gene expression signatures tested during model development in NeoALTTO, with gene names (entrez ID) and coefficients included.
- **Supplementary data 40**: Gene expression signatures tested in NeoALTTO and CALGB 40601 when comparing prognostic groups, with gene names (entrez ID) included.
